# The ubiquitin proteasome system in glia and its role in neurodegenerative diseases

**DOI:** 10.3389/fnmol.2014.00073

**Published:** 2014-08-08

**Authors:** Anne H. P. Jansen, Eric A. J. Reits, Elly M. Hol

**Affiliations:** ^1^Department of Cell Biology and Histology, Academic Medical CenterAmsterdam, Netherlands; ^2^Department of Translational Neuroscience, Brain Center Rudolf Magnus, University Medical Center UtrechtUtrecht, Netherlands; ^3^Netherlands Institute for Neuroscience, Institute of the Royal Netherlands Academy of Arts and SciencesAmsterdam, Netherlands; ^4^Swammerdam Institute for Life Sciences, Center for Neuroscience, University of AmsterdamNetherlands

**Keywords:** astrocytes, microglia, oligodendrocytes, gliosis, ubiquitin proteasome system, neurodegenerative diseases

## Abstract

The ubiquitin proteasome system (UPS) is crucial for intracellular protein homeostasis and for degradation of aberrant and damaged proteins. The accumulation of ubiquitinated proteins is a hallmark of many neurodegenerative diseases, including amyotrophic lateral sclerosis, Alzheimer’s, Parkinson’s, and Huntington’s disease, leading to the hypothesis that proteasomal impairment is contributing to these diseases. So far, most research related to the UPS in neurodegenerative diseases has been focused on neurons, while glial cells have been largely disregarded in this respect. However, glial cells are essential for proper neuronal function and adopt a reactive phenotype in neurodegenerative diseases, thereby contributing to an inflammatory response. This process is called reactive gliosis, which in turn affects UPS function in glial cells. In many neurodegenerative diseases, mostly neurons show accumulation and aggregation of ubiquitinated proteins, suggesting that glial cells may be better equipped to maintain proper protein homeostasis. During an inflammatory reaction, the immunoproteasome is induced in glia, which may contribute to a more efficient degradation of disease-related proteins. Here we review the role of the UPS in glial cells in various neurodegenerative diseases, and we discuss how studying glial cell function might provide essential information in unraveling mechanisms of neurodegenerative diseases.

## THE UBIQUITIN PROTEASOME SYSTEM

Protein homeostasis is essential for proper function of a cell; therefore both protein synthesis and degradation are tightly regulated. Although there is a complex interplay between the two systems, in general, there are two main machineries involved in protein degradation: autophagy and the ubiquitin proteasome system (UPS). Autophagy involves lysosomal degradation by the formation of intracellular vesicles (autophagosomes) and is subdivided in chaperone-mediated autophagy (CMA), microautophagy, and macroautophagy. In CMA, chaperone proteins bind specifically to a KFERQ domain in a cytosolic protein, afterward it is internalized and degraded. Microautophagy is the direct lysosomal digestion of cytoplasmic content, which is trapped by random invagination of the lysosomal membrane. Macroautophagy functions as bulk degradation of mainly long-lived proteins, protein aggregates and organelles, and involves autophagosome formation to isolate cytoplasmic proteins. The UPS selectively targets individual proteins, including short-lived, damaged or defectively folded proteins, which accounts for about 80–90% of all intracellular proteins (Rock et al., 1994; Lilienbaum, 2013).

**FIGURE 1:**
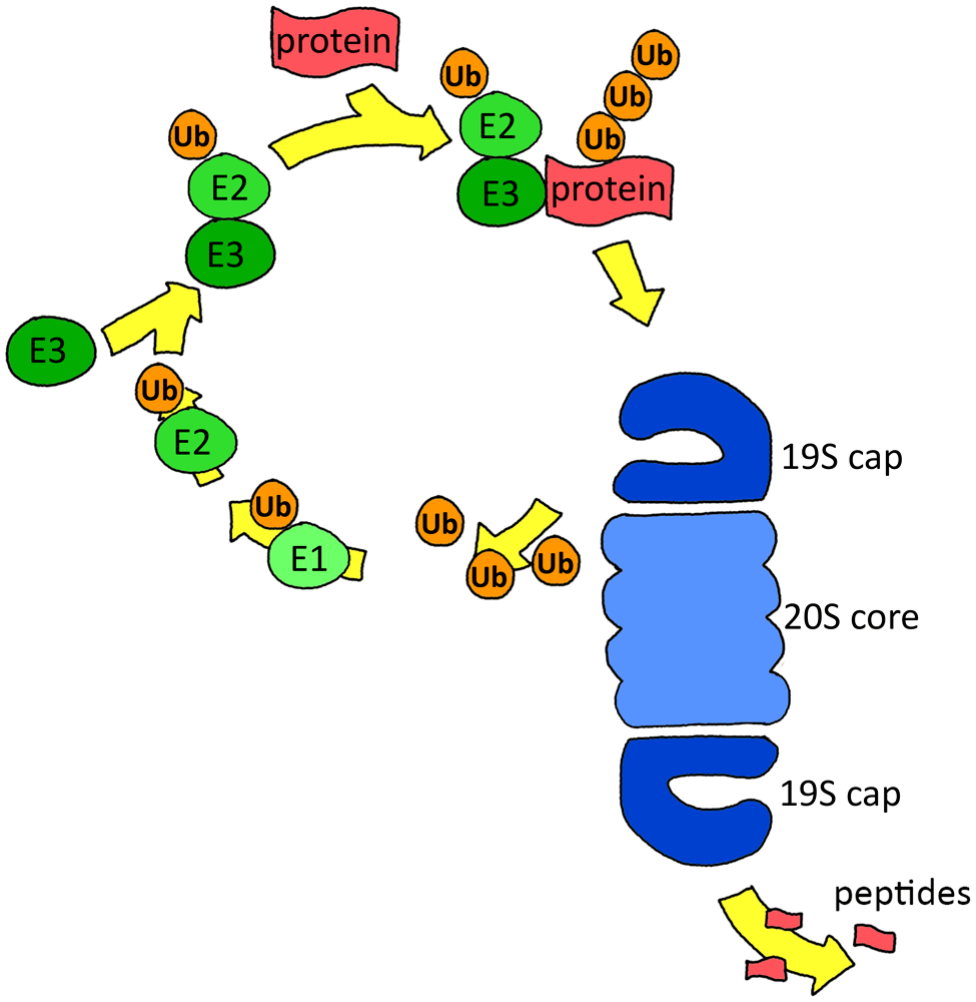
**Scheme of the ubiquitin proteasome system.** Proteins are targeted for degradation by ubiquitination via E1, E2, and E3 enzymes. The proteasome recognizes and removes the ubiquitination signal, unfolds the protein and finally degrades it.

The UPS consists of two key components: the ubiquitination system, which selects and targets proteins towards degradation by ubiquitinating them, and the proteasome, a multimeric protein complex that actually performs the degradation. Protein degradation is a tightly regulated process: before a protein is cleaved by the proteasome, an elaborate process of selection and targeting has taken place exerted by the ubiquitin (Ub) system. This system mediates the conjugation of Ub, a small 76-amino-acid-long modifier. Binding of Ub to the target protein takes place in a three-step reaction. First, Ub is linked to an Ub-activating enzyme (E1) in an ATP-dependent manner. Subsequently, the activated Ub is transferred to an E2 conjugating enzyme, followed by attachment of E2 to a specific E3 ubiquitin ligase enzyme that binds the target protein. Lastly, Ub is transferred by the E2 enzyme to the target protein (**Figure 1**). Ub itself can be ubiquitinated at one of its seven lysine residues, resulting in various polyubiquitin chain types that each has its own specific signal function. Regulation of protein degradation is also mediated by deubiquitinating enzymes (DUBs) that can reverse ubiquitination by removing Ub residues of mono- or polyubiquitinated proteins (Glickman and Ciechanover, 2002; Lilienbaum, 2013).

Polyubiquitinated proteins are generally degraded by the 26S proteasome, which consists of a barrel-shaped 20S core that is the actual protease and a 19S regulatory complex that recognizes and unfolds the ubiquitinated substrate (**Figure 1**). The 20S core consists of two rings of seven α subunits, flanking two rings of seven β subunits. Three of these seven β subunits are catalytically active, with a caspase-like (β1), trypsin-like (β2), or chymotrypsin-like (β5) activity that cleave after negatively charged, positively charged, and hydrophobic amino acids, respectively (Lilienbaum, 2013). The 19S regulatory complex recognizes and unfolds the ubiquitinated substrate and guides it into the 20S core. The 19S complex consists of 19 individual proteins, of which six are ATPases of the AAA family (Baumeister et al., 1998). Binding of ATP is necessary for assembly of the 20S core with the 19S cap, subsequently ATP hydrolysis is required for protein unfolding, while for the next steps, gate opening, translocation and degradation, only ATP binding is required (Smith et al., 2005).

Next to the 19S complex, also other regulatory proteasome activators (PA) exist, including 11S (PA28), PI31, and PA200, which bind the 20S core in an ATP-independent way (Lilienbaum, 2013). PA28αβ expression is induced upon secretion of interferon gamma (IFNγ) and plays a role in processing peptides for MHC class I antigen presentation (Realini et al., 1994; Sijts and Kloetzel, 2011). When PA28αβ binds to 20S core, the activities of all β-subunits are increased, which is probably due to an increased accessibility rather than alterations within the active sites themselves. In contrast to PA28αβ, the exact mechanism that PA28γ, which is only expressed in the nucleus, uses to exert its function is not known (Rechsteiner and Hill, 2005).

IFNγ not only induces different PA caps but also induces the expression and incorporation of the proteasomal immuno-subunits β1i (LMP2), β2i (Mecl-1), and β5i (LMP7) that substitute β1, β2, and β5, respectively. With this replacement, the chymotrypsin-like activity increases and the immunoproteasome shows an altered cleavage pattern, resulting in different peptides being generated that are subsequently presented by MHC class-I molecules to the cells of the immune system (Sijts and Kloetzel, 2011; Basler et al., 2013).

## GLIAL CELLS

**FIGURE 2:**
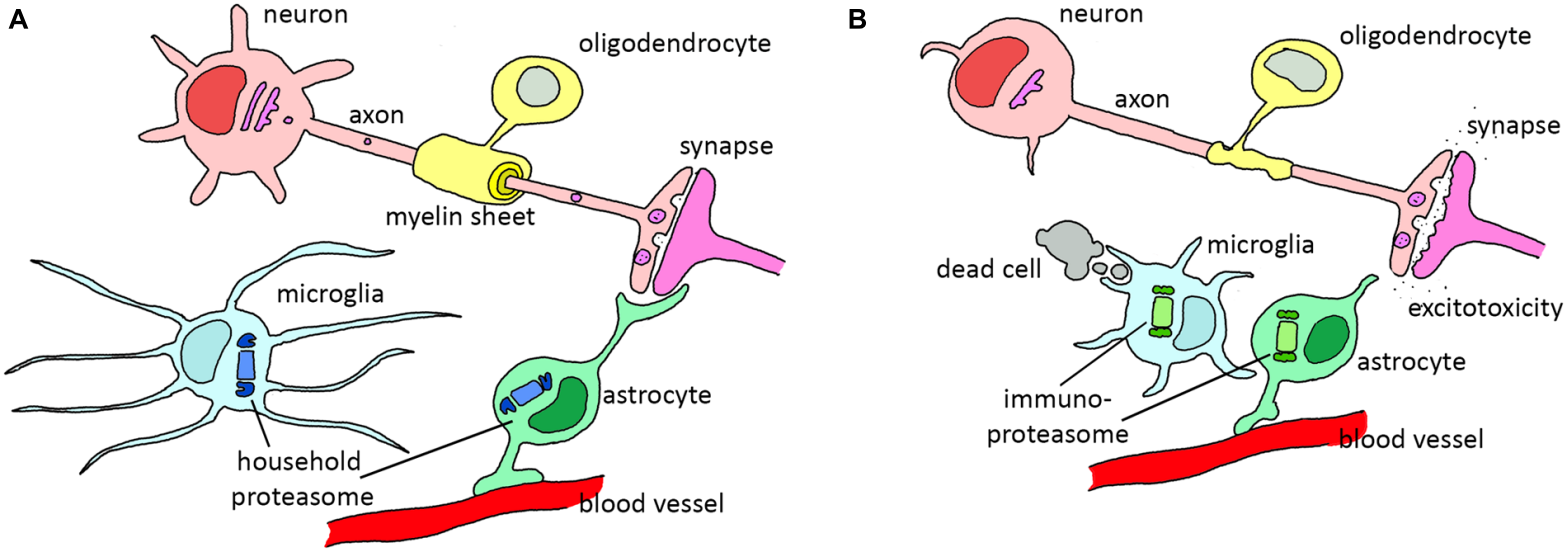
**The role of various glial cells in the healthy and diseased CNS. (A)** In the healthy situation, astrocytes support neurons by filtering nutrients from the blood and modulating synapses, oligodendrocytes wrap axons in isolating myelin sheets and microglia scan the environment for dead cells or invading pathogens. **(B)** During reactive gliosis in neurodegenerative diseases, oligodendrocytes and astrocytes lose their normal support function. Astrocytes decrease glutamate uptake from the synapse, leading to excitotoxicity. Microglia become activated and phagocytize dead cells and start to secrete pro-inflammatory cytokines that activate astrocytes. Both reactive astrocytes and activated microglia upregulate immunoproteasome expression. In particular diseases, demyelination of the axons is observed due to oligodendrocyte dysfunction.

Neuroglia were first described by the German pathologist Rudolf Virchow already more than 150 years ago. For a long time these cells were considered as nerve glue, a kind of connective tissue holding the brain together. In contrast to neurons, glia are not electrically excitable. This made it difficult to study these cells, and as a consequence, most neuroscientific research was focused on neuronal function. When sophisticated molecular tools became available which made it possible to study the physiology of glial cells, this has led to a change in the neurocentric view of neuroscientists. During the last decades, it has become evident that glial cells are essential for proper neuronal function, are actively involved in neuronal communication, and form the immune system of the brain. As diverse as different glial cells are in morphology and origin, as diverse they are likely in their function. They take care of the general homeostasis in the brain, insulate neurons, and protect against pathogenic invaders (Kettenmann and Verkhratsky, 2008). The ratio between neurons and glia in the human central nervous system (CNS) is about 1:1, with oligodendrocytes being the most abundant type of glial cells (75.6%), followed by astrocytes (17.3%) and microglia (6.5%) in human male brains (Pelvig et al., 2008). Below, the most important glial cell types and their functions are described (**Figure 2**).

### ASTROCYTES

Astrocytes perform a variety of crucial tasks in the CNS. Similar to neurons, astrocytes are of ectodermal origin. During the first stages of embryogenesis, the first astrocyte precursor cells arise from neural stem cells. These radial glia function as neural progenitors and form the scaffold that is used by immature neurons to migrate toward their final location. In a later stage, these cells give rise to the progenitors of oligodendrocytes as well as to the different kinds of astrocytes in the brain. The astrocyte population is broad and heterogeneous and the classification of subtypes is in its initial stage. Astrocytes contact multiple blood vessels with their endfeet, and they connect to neighboring astrocytes via gap junctions. Besides, astrocytes envelope multiple synapses and fibrous astrocytes in the white matter contact several nodes of Ranvier, which are the small gaps in myelinated axons (Sofroniew and Vinters, 2010; Molofsky et al., 2012). Subventricular astrocyte-like cells are the stem cells in the brain. The progeny of these cells migrate through the rostral migratory stream into the olfactory bulb, and differentiate into interneurons. When isolated and brought in culture, they can differentiate in both neurons and glia thus exhibiting multipotent properties (Mamber et al., 2013). These cells are even still present in aged brains, suggesting regenerative potential until late in life (Van Den Berge et al., 2010).

In the mature brain, astrocytes influence synaptic transmission directly by the release of gliotransmitters including glutamate, ATP, GABA, and D-serine, although it is not fully understood how the cells release these transmitters. Astrocytes also respond to neurotransmitters, which leads to a surge in calcium. Although astrocytes do have voltage-gated channels (Pappalardo et al., 2014), they are not able to respond with an increase in calcium in a millisecond time scale. Hence, astrocytes play a modulatory role: they are likely to influence synaptic communication of the many neurons in their domain. Astrocytes can also regulate synaptic transmission by mediating homeostasis of ions, pH and (neuro)transmitters. A well-known function of astrocytes is uptake of glutamate from the synaptic cleft via glutamate transporter Glt-1. Moreover, astrocytes contain transporters for the neurotransmitters GABA and glycine, for K^+^ ions, bicarbonate, water and monocarboxylic acid (Sofroniew and Vinters, 2010). Recently, it has been shown that astrocytes are able to influence synapses also by synaptic pruning both in the developing and in the adult mouse brain. This phagocytosis of synapses is dependent on neuronal activity and is mediated via the Mertk and Megf10 pathways. Thus, astrocytes actively contribute to activity-dependent synapse elimination and CNS remodeling (Chung et al., 2013). Next to the phagocytosis of synapses, activated astrocytes are able to phagocytize amyloid β deposits *in vitro* and *in situ* (Wyss-Coray et al., 2003).

Furthermore, astrocytes form a key compartment of the blood brain barrier (BBB); they are not only involved in induction and development of the BBB, they also regulate BBB permeability (Abbott, 2002). Astrocytes are connecting the blood vessels with many neuronal perikarya, axons and synapses. Therefore, astrocytes are the ideal cells to make an important contribution to the energy supply of the brain; for example by taking up glucose from the blood via their glucose transporters.

In conclusion, astrocytes are a heterogeneous group of glial cells that exert numerous tasks essential for proper neuronal function. They are indispensable in neurodevelopment, synaptic communication and brain homeostasis. As a consequence, loss or change in astrocyte function can contribute to pathogenesis of neurodegenerative diseases. A clear example is the leukodystrophy Alexander’s disease, which is caused by a mutation in the astrocyte specific gene GFAP that leads to astrocyte pathology and dysfunction (Brenner et al., 2001; Sosunov et al., 2013).

### OLIGODENDROCYTES

Oligodendrocytes are the myelinating cells of the brain, and form an insulating myelin sheath around the axonal segments of neurons. Like astrocytes, oligodendrocytes are of ectodermal origin; they arise from the neural stem cells in the neuroepithelium during early embryogenesis. The onset of myelination, as well as the selection of axons that require myelination, are both tightly regulated. Neuronal activity and degree of differentiation, together with several surface receptors (e.g., LINGO-1), influence the brain area and specific axons that are myelinated (Baumann and Pham-Dinh, 2001; Bradl and Lassmann, 2010). As oligodendrocytes have only a short time window wherein they are capable of myelination, this provides temporal control during early differentiation as well (Baumann and Pham-Dinh, 2001). A single oligodendrocyte can enwrap multiple axons, while a single axon can have adjacent myelin sheets belonging to different oligodendrocytes. Myelination of axons is important for a high-speed, reliable conduction of electrical signals between neurons. The myelinated fibers contain small gaps named nodes of Ranvier that are important for the fast signal conduction, so the action potential can jump from node to node. During the peak of myelination, about three times the weight of the oligodendrocyte is produced in myelin each day. Small changes in protein homeostasis will therefore have huge consequences for protein production and quality control, which can result in misfolding and accumulation of proteins and cause dysfunctional oligodendrocytes and eventually demyelination. Illustratively, transgenic rat overexpressing the oligodendrocyte proteolipid protein showed oligodendrocyte apoptosis and dysmyelination (Baumann and Pham-Dinh, 2001; Bradl and Lassmann, 2010). Axons can be remyelinated by new oligodendrocytes arising from the NG2^+^ cells that are stimulated to divide and differentiate by activated microglia and astrocytes. However, the resulting myelin sheath is thinner, possibly due to the lack of stimulating growth factors that were initially secreted by the growing axon. Oligodendrocyte dysfunction is directly detrimental for neuronal function and leads to neurodegeneration in the α-synucleinopathy multiple system atrophy (MSA), but also in multiple sclerosis and other leukodystrophies (Baumann and Pham-Dinh, 2001; Kuzdas et al., 2013).

### MICROGLIA

Microglia primarily function as the immune cells of the CNS. In contrast to astrocytes and oligodendrocytes, microglia are from mesodermal origin (Ginhoux et al., 2010) and are derived from hematopoietic stem cells in the yolk sac early in embryonic development (Alliot et al., 1999). Although microglia are mostly known for their function in the immune response under disease conditions, they also play an important role in CNS homeostasis. During neuronal development, microglia regulate brain plasticity, by controlling synapses (Kettenmann et al., 2013). Next to phagocytizing apoptotic cells and pruning synapses during development, microglia are actively involved in promoting apoptosis, which is also an important aspect of neuronal development (Kettenmann et al., 2013). In the adult brain, microglia also phagocytize dead neurons and express several receptors for neurotransmitters, -peptides, and -modulators, thereby sensing (changes in) neuronal activity. Under healthy conditions, microglia have a small nucleus and long, thin, very motile processes, and although called “resting microglia,” these cells are constantly monitoring the environment and when dying neurons are detected, microglia become activated. Subsequent phagocytosis is a tightly regulated process involving multiple receptors like CD36, lectin, and integrin receptors (Kettenmann et al., 2013).

Next to neuronal damage, invading pathogens are detected and cleaned up by microglia (Aloisi, 2001). To recognize and eliminate foreign entities, a variety of pattern recognition receptors are expressed including complement receptor 3 and toll-like receptors (TLR) 2, 4, and 9 that promote phagocytosis and recognize bacterial lipopolysaccharide (LPS; TLR2, 4) or DNA (TLR9). These cytokines are secreted by CNS associated macrophages, astrocytes and by other microglia as a paracrine activation mechanism. TNF-α, IL-1, and IFNγ are pro-inflammatory cytokines that activate the immune functions of microglia including phagocytosis, cytokine production and antigen presentation. The immune response is regulated by anti-inflammatory cytokines such as IL-10, TGF-β, which downregulate the expression of proinflammatory cytokines, reactive oxygen species (ROS), chemokines and other molecules associated with phagocytosis in microglia (Aloisi, 2001; Tambuyzer et al., 2009).

## GLIA IN PATHOLOGY

Early pathological studies showed astrogliosis and microglial proliferation in damaged brain tissues in several neurodegenerative diseases including Huntington’s disease (HD; Faideau et al., 2010), Alzheimer’s disease (AD; Sheng et al., 1997; Mrak, 2009), but also in inflammatory diseases, brain trauma, ischemia and infection (Gray et al., 1996; Tambuyzer et al., 2009; Amor et al., 2013; Pekny et al., 2014). Since microglia sense neuronal damage, they might be the initiators of the glial reaction to neuronal pathology, which is called neuroinflammation (reactive gliosis). Activated microglia have been shown to secrete cytokines that induce astrocyte reactivity. This is a delayed reaction; microglia are sensitive for the smallest pathological changes in the CNS, but only when the damage is severe enough this will lead to astrogliosis. Astrogliosis is characterized by increased expression of the astrocyte-specific intermediate filament GFAP (Pekny et al., 2014) and vimentin (Parpura et al., 2012). Astrocytes express cytokine receptors for, e.g., IL-1, IL-6, and TNF-α, and microglia might disturb normal astrocyte function by activating astrocytes via these receptors. For example, TNF-α affects glutamate transmission directly by inhibiting expression of astrocytic glutamate transporters (Cumiskey et al., 2005). As a result of activation, astrocytes release cytokines that can in turn influence microglial function (Amor et al., 2013). Hereby a feedback loop is created, in which factors from both astrocytes and microglia regulate each other (Zhang et al., 2010).

It is unclear whether glial activation under pathological circumstances is beneficial or detrimental. Activation of astrocytes is initially meant to protect neurons by forming a protective border around the damaged site (Pekny et al., 2014), but loss-of-function of astrocytes such as reduced glutamate uptake may also result in neuronal dysfunction (Cumiskey et al., 2005). LPS-induced microglial activation is associated with subsequent astrocyte and oligondendrocyte impairment and demyelination. LPS induction of microglia caused a decrease in expression in several astrocytic proteins that are important for their normal function (e.g., Aqp4, a water transport channel). Astrocytes connect to each other and to oligodendrocytes via gap junctions, and activated astrocytes show reduced expression of connexins (30 and 43) that mediate these junctions. As ions, water and osmolites are exchanged via these connexins, loss of this connection leads to decreased oligodendrocyte function and subsequent demyelination and neuronal impairment (Sharma et al., 2010). In astrocytes, Dicer ablation causes changes in the transcriptome resulting in the upregulation of many immature/reactive astrocyte genes, while astrocytic genes related to mature astrocyte functions are downregulated (e.g., GLT-1). The alterations in astrocyte phenotype occur already before neuronal deficits were perceived, underlining the importance of proper astrocyte function in the brain (Tao et al., 2011).

## A DIVERGENCE IN UPS BETWEEN NEURONS AND GLIA

Since glia are essential for proper neuronal function, disturbances in glial function can lead to excitotoxicity and neurodegeneration. However, in neurodegenerative diseases with protein inclusions (proteinopathies), neurons appear to be most vulnerable, as protein aggregation and cell degeneration is mainly observed in neurons. Possibly these differences are due to dissimilarities in their protein homeostasis. Indeed, distinct cell types show divergent protein synthesis and degradation (Vilchez et al., 2012). In addition, differences may exist in UPS levels and activity between neurons and the various glial cells. The UPS is the main protein complex involved in the degradation of oxidized proteins, and oxidation of nucleic acids, lipids and proteins is associated with aging and neurodegenerative diseases (Mariani et al., 2005). Proteasome inhibition causes an increase in nucleic acid oxidation in both primary neurons and astrocytes; however, this increase is much larger in neurons. This suggests that neurons are more sensitive to proteasome inhibition, or are more prone to oxidative stress, which is associated with neurodegenerative diseases like AD and Parkinson’s disease (PD; Ding et al., 2004). The UPS appears to be less active in neurons in comparison to white matter glia (Tydlacka et al., 2008). Intriguingly, in response to cytokines such as TNF-α, IL-2, G-CSF, and IFNγ, neurons upregulate the Ub-like protein ubiquitin D (or Fat10; Lisak et al., 2011), which is able to target long-lived proteins to the proteasome. Conversely, both astrocytes and microglia failed to upregulate Fat10 upon cytokine stimulation (Hipp et al., 2005), also indicating that the UPS is regulated in neurons in another way. In contrast to most other neurodegenerative diseases, in intranuclear inclusion body disease (INIBD) the largest proportion of aggregates are found in glial cells (about 5% in glial cells, compared to 1% in neurons). Here, most aggregates are present in astrocytes, and a smaller proportion in oligodendrocytes. All aggregates are positive for Ub, however, glial cells seem to have a significantly smaller proportion of aggregates positive for the Ub-like proteins NEDD8, NUB1, and SUMO1 than neurons. This slightly dissimilar composition of the aggregates can be explained by either variations in protein expression or protein recruitment toward the aggregates in the cell types (Mori et al., 2012). In conclusion, neurons and glia are differently reacting to proteasome inhibition and cytokines. Besides, presence of UPS components in inclusion bodies seem to diverge between neurons and glia, which could be indications why neurons are more vulnerable in neurodegenerative diseases.

## UPS IN GLIA IN RELATION TO NEURODEGENERATIVE DISEASES

The tight balance between protein synthesis and degradation is essential for cellular function. In the brain, disturbances in either of the two cause the accumulation and aggregation of short-lived and misfolded proteins. In aging neurons, UPS activity has been shown to be decreased (Tydlacka et al., 2008), accordingly, age-related alterations in proteasomal activity are implicated in various neurodegenerative diseases (Tydlacka et al., 2008; Tashiro et al., 2012; Lin et al., 2013; Orre et al., 2013). It remains to be examined in more detail whether and how proteasome levels and activity differ between neurons and glia, which would obviously affect the capacity of cells to maintain proper protein homeostasis. In many diseases protein aggregates are hallmarks that are clearly visible in neurons, and are indicative for neuronal dysfunction. However, aggregates are also described in glial cells, for example in HD (Shin et al., 2005; Tong et al., 2014) and amyotrophic lateral sclerosis (ALS; Bruijn et al., 1997), although they are usually smaller or less abundant. The discrepancy between the occurrence of neuronal and glial inclusions implies that the UPS system is more efficient to remove aggregation-prone proteins in glia. Yet, while glia might be better able to handle these proteins, this does not mean that glia are not affected. Mainly astrocytes and microglia react to the increase of aggregated proteins in the brain and show altered function, thereby contributing to neuronal dysfunction. Many studies have shown that neuronal UPS dysfunction plays an important role in several neurodegenerative diseases. The first indications were found already more than 25 years ago, when components of the UPS were found in inclusions of several neurological diseases including PD, Pick’s disease but also in Rosenthal fibers in astrocytomas (Lowe et al., 1988). Depletion of 26S proteasome in mice neurons led to significant neurodegeneration and inclusion body formation (Bedford et al., 2008). Although the depletion was restricted to neurons, changes in glia were observed as astrocytes showed an increase in GFAP and vimentin protein expression, indicative of reactive gliosis (Elkharaz et al., 2013). Interestingly, astrocytes themselves seem to be less sensitive for proteasome inhibition. It has recently been suggested that this is due to the high expression of the heat shock protein HSP25 (Goldbaum et al., 2009). Although inhibition of proteasomes by the reversible proteasome inhibitor MG-132 caused aggresome formation and cytoskeletal disturbances in cultured primary astrocytes, cell viability was only diminished by 20%. Besides, cytoskeletal disturbances appeared to be reversible in astrocytes, whereas in oligodendrocytes proteasome inhibition massively induced apoptosis. This effect might be caused by the induction of the HSP25, which is expressed at a much higher level in astrocytes and interacts with all three types of cytoskeletal filaments in astrocytes. As downregulation of this protein indeed resulted in fragmentation of actin networks, these results suggest a protective role of HSP25 in the astrocyte cytoskeleton during proteasome inhibition (Goldbaum et al., 2009). Proteasome inhibition does not only change cell viability, it has also been shown to decrease intermediate filament transcription in astrocyte cell lines. Treatment with proteasome inhibitors epoxomicin and MG-132 decreased the mRNA levels of the astrocyte-specific intermediate filament GFAP in different astrocyte cell lines. Similarly, vimentin and nestin expression were significantly decreased upon proteasome inhibition in astrocytes, but not in neuronal cells. Moreover, rat brains treated with proteasome inhibitors through a cannula showed less astrogliosis around the cannula. It appeared that the proteasome can affect GFAP promotor activity by the degradation of its transcription factors, thereby linking UPS activity directly to astrogliosis (Middeldorp et al., 2009).

Cultured primary oligodendrocytes seem to be much more sensitive to proteasome inhibition when compared to astrocytes. Treatment of these cells with MG-132 caused oxidative stress, mitochondrial dysfunction and apoptosis after 18 h (Goldbaum et al., 2006). While inhibition of the 26S regulatory subunit 7 (RPT1, part of 19S) specifically reduced pro-inflammatory cytokine secretion in LPS-stimulated cultured microglia (Bi et al., 2012), in general also microglia showed a decreased survival and an increase in pro-inflammatory response following proteasome inhibition, including upregulation of nitric oxide and TNF-α secretion (Kwon et al., 2008). LPS induction also triggers the secretion of these cytokines by microglia, while intracellularly the pro-inflammatory NF-κB pathway is upregulated. This process is controlled by UPS components, with E3 ligase RING finger protein 11 (RNF11) as one of the key negative regulators of the NF-κB pathway in microglia (Dalal et al., 2012). In neurons and microglia, IFNγ caused induction of the immunoproteasome by replacing the constitutive subunits β1, β2, and β5 with the inducible immuno subunits β1i, β2i, and β5i and the association with the regulatory complex PA28αβ. Synergistically, LPS-induced neuroinflammation can aggravate neurodegeneration that is triggered by proteasome inhibition (Stohwasser et al., 2000; Pintado et al., 2012).

Since both proteasome inhibition and neuroinflammation are associated with neurodegenerative diseases, studying the interplay between those two will provide new insights in neurodegenerative disease mechanisms. In the following section, proteasome dysfunction and its effect on glia in several age-related neurodegenerative diseases are described and in **Table 1** an overview of the literature on a wider range of neurodegenerative diseases is given.

**Table 1:** Overview of published data on the role of glia and the UPS in several neurodegenerative diseases.

Disease	Aggregation in glia	UPS changes
Alzheimer’s disease and other tauopathies	Tau in astrocytes and oligodendrocytes (Ferrer et al., 2014)	Immunoproteasome induction in astrocytes and microglia (Mishto et al., 2006; Nijholt et al., 2011; Orre et al., 2013)
Huntington’s disease	Htt in astrocytes (Shin et al., 2005; Tydlacka et al., 2008; Tong et al., 2014)	Immunoproteasome induction in neurons (Díaz-Hernández et al., 2003)
Parkinson’s disease and other α-synucleopathies	α-syn in oligodendrocytes and possibly astrocytes (Casarejos et al., 2009; Stefanova et al., 2012; Pasanen et al., 2014)	No changes
Amyotrophic lateral sclerosis	SOD1 and C9orf72 in astrocytes, microglia and oligodendrocytes (Bruijn et al., 1997; Forsberg et al., 2011; Mizielinska et al., 2013)TDP-43 in oligodendrocytes (Brettschneider et al., 2013)	Immunoproteasome induction in astrocytes, microglia, oligodendrocytes and neurons. (Puttaparthi et al., 2007; Cheroni et al., 2009; Bendotti et al., 2012)TDP-43 aggregates are Ub positive (Zhang et al., 2008)
Multiple sclerosis	No aggregates	Immunoproteasome induction in astrocytes, microglia, neurons and lymphocytes (Mishto et al., 2010; Zheng et al., 2012; Popescu et al., 2013; Bellavista et al., 2014)
Alexander’s disease	GFAP and TDP-43 in astrocytes in Rosenthal fibers (Brenner et al., 2001; Cho and Messing, 2009; Sosunov et al., 2013; Walker et al., 2014)	Impaired proteasome function in astrocytes (Tang et al., 2006, 2010)

### ALZHEIMER’S DISEASE

Alzheimer’s disease is the most prevalent form of dementia and is characterized by irreversible memory loss and impaired cognitive functions. Magnetic resonance imaging (MRI) scans of AD patient brains reveal atrophy and an enlargement of the ventricles caused by neuronal shrinkage or cell loss, and loss of (synaptic) connections is thought to be the main contributor to the cognitive and memory impairments (Bozzali et al., 2011). Neuropathological hallmarks of AD are senile plaques and neurofibrillary tangles. Neurofibrillary tangles are formed by the hyperphosphorylated microtubule-associated protein tau and are located mainly inside neurons, while extracellular the senile plaques consist of aggregates formed by the peptide amyloid β (Aβ). Over the last two decades, the concept of a neuroinflammatory involvement in AD has been established. This started in 1986 with the discovery of plaque-associated reactive microglia, and additional studies described the association of both activated microglia and astrocytes with Aβ plaques expressing pro-inflammatory markers (Rozemuller et al., 1986; Griffin et al., 1989; Mrak, 2009). Astrocytes change their phenotype when contacting extracellular Aβ, and their response includes upregulation of glial fibrillary acidic protein (GFAP), vimentin and S100β (Griffin et al., 1989; Kamphuis et al., 2012). In AD, both astrocytes and microglia play an important role in clearance of Aβ plaques (Wyss-Coray et al., 2003; Mandrekar et al., 2009; Mulder et al., 2014). Besides, attenuation of astrocyte activation leads to a higher plaque load and increased microglial activation, probably as a compensatory mechanism (Kraft et al., 2013). A loss in metabolic function of activated astrocytes worsens the disease, and upregulation of GFAP has been shown to correlate with downregulation of the astrocyte-specific glutamate transporter EAAT2 (Simpson et al., 2010; Yan et al., 2013).

Changes in the UPS pathway have been associated with AD in several studies. Ub is accumulating in both plaques and tangles (Chu et al., 2000). Next to the regular Ub protein in the brains of AD and Down syndrome patients, both having the plaque pathology, a unique form of ubiquitin B can be found. This protein originates from a frameshift mutation in the mRNA and leads to a protein with an aberrant C-terminus, called UBB+1. It can be detected in all AD and Down syndrome patients, but in none of the non-demented controls (van Leeuwen et al., 1998). Because of the absence of the carboxy-terminal Gly-76, UBB+1 cannot ubiquitinate other proteins, but is itself efficiently ubiquitinated. However, the proteasome seems unable to degrade large amounts UBB+1, leading to proteasome inhibition (Lam et al., 2000; van Tijn et al., 2007). In addition, UBB+1 expression also changes the immune response in astrocytes upon TNF-α and IFNγ treatment as secretion of the chemokines CCL2 and CXCL8 is increased dramatically. This is probably due to an upregulation of several proteins in the NF-κB and JNK pathways, suggesting that a disruption in the UPS directly influences pro-inflammatory signaling in astrocytes and *vice versa* (Choi et al., 2013).

Importantly, alterations in the UPS can influence the degradation of Aβ in both neurons and astrocytes. Although mainly the neuronal viability is affected in response to increasing Aβ levels, also astrocytes showed a similar upregulation in UPS related proteins and a decrease in proteasome activities upon Aβ treatment as neurons (Lopez Salon et al., 2003). Treatment with Aβ oligomers decreased proteasome activity *in vitro* and in mouse brain lysates, and a lower proteasome activity was also observed in lysates of several brain areas of AD patients (Gregori et al., 1995; Keller et al., 2000; Tseng et al., 2008; Zhao and Yang, 2010). However, these measurements were mainly performed in whole brain homogenates or in (neuronal) cell lines, and may therefore fail to see alterations in immunoproteasome levels and activity in glial cells since that was not investigated in these studies. More recently, it was shown that Aβ treatment led to an increase in proteasome activity in both cultured neurons, astrocytes and microglia. In addition, it was discovered that both mRNA and protein levels of immunoproteasome subunits β5i and β1i were upregulated in reactive astrocytes and microglia around plaques in AD mice and human AD patient material, whereas in AD mice the levels of β2i were similar to controls. Consistently, the activity of all immunoproteasome subunits was increased with increasing plaque load in both AD mice and in post-mortem material of human AD patients (Nijholt et al., 2011; Orre et al., 2013). These results are in line with earlier data showing that β1i expression levels in neurons and astrocytes were increased with age, and in the brain areas most affected in AD (Mishto et al., 2006). While it becomes clear that especially immunoproteasome activity is increased in glia during AD, it remains to be examined whether these changes are beneficial or not.

### PARKINSON’S DISEASE

Parkinson’s disease is after AD the most common neurodegenerative disease with an incidence of 0.3% in the total population. The incidence increases with age leading to 4% of people above the age of 80 suffering from this mostly idiopathic movement disorder (Imai et al., 2000; de Lau and Breteler, 2006). The etiology of the disease is not known, although there are some familial cases caused by mutations in the genes of, e.g., alpha-synuclein (α-syn) and E3 ligase parkin. Also mutations in UCH-L1, a Ub c-terminal hydrolase, have been associated with PD but this finding is controversial (Hardy et al., 2009). In PD, the dopaminergic neurons in the substantia nigra pars compacta degenerate, which is accompanied with eosinophillic intracytoplasmatic inclusions known as Lewy bodies (LBs) and gliosis. While microgliosis is dominantly present in PD, reactive astrogliosis is virtually absent (Mirza et al., 1999; Van Den Berge et al., 2010; Halliday and Stevens, 2011). Although they do not change their phenotype, astrocytes are affected by α-syn accumulation. Astrocytes are able to take up α-syn released from neurons, and subsequently secrete pro-inflammatory cytokines (TNF-α and CXCL1), thereby activating microglia (Lee et al., 2010). These results were confirmed in a study using a mouse model expressing α-syn in astrocytes only, which caused similar disease symptoms, astrocyte functional loss and activated microglia. As the pro-inflammatory response of these cells caused neurodegeneration (Gu et al., 2010), this suggests that glial dysfunction plays a significant role in the etiology of PD.

A role for the UPS in PD was first described in familial PD when it was discovered that mutations in the protein parkin were associated with familial PD. Parkin functions as a Ub ligase in association with proteasomal degradation (Imai et al., 2000). A subset of the mutations in parkin, but also post-translational modifications of the protein, have been shown to cause a loss of function of the E3 ligase, and have been associated with UPS impairment and abrogation of the neuroprotective effects of parkin (Tsai et al., 2003; Yamamoto et al., 2005; Ali et al., 2011). In addition, Lewy bodies are mainly composed of α-syn and cytoskeletal proteins, but next to these proteins Lewy bodies contain the UPS related proteins parkin and Ub (Shults, 2006). Induced mutant α-syn expression was reported to decrease proteasome activity in cultured cells and a decreased proteasome activity was detected in the substantia nigra of PD patients (Tanaka et al., 2001; McNaught et al., 2003). These findings are in contrast with a study that showed that overall proteasome activity was not decreased in brain regions with Lewy body pathology (Tofaris et al., 2003). The discrepancy between these results can be explained by the technique that was used to measure proteasome activity in these studies. The AMC-peptides are rather non-specific since also other proteases can cleave these peptides, thereby influencing the results. Therefore, the exact role of UPS in PD is still under debate. Cultured parkin knock-out neurons appeared to be more resistant to mild proteasome inhibition than wild type neurons due to upregulation of anti-oxidant scavenger protein glutathione and autophagy-related proteins. In contrast, parkin knock-out glia (mixed population, mainly astrocytes) were more susceptible to epoxomicin-induced cell death than wild type glia, suggesting that parkin dysfunction in PD mainly affects glia. In contrast to neurons, this effect on parkin knock-out glia was associated with lower levels of glutathione, a decreased HSP70 response, and an increase in poly-ubiquitinated proteins, all contributing to glial dysfunction. Besides, conditioned medium of parkin knock-out glia was less neuroprotective. So proteasome inhibition causes glial dysfunction and is therefore possibly the reason for the pathology in Parkin knock-out mice (Solano et al., 2008; Casarejos et al., 2009). Correspondingly, proteasome inhibitors had deleterious effects in mice expressing α-syn only in oligodendrocytes. The mice showed motor symptoms and severe degeneration in the nigrostriatal pathway caused by myelin disruption and demyelination in oligodendrocytes that had accumulated α-syn fibrils in their cytoplasm (Stefanova et al., 2012). Overall in PD, inhibition of the UPS system seems to contribute to glial dysfunction, thereby also affecting neuronal function. However, similar to AD the exact mechanism causing proteasome changes and neurodegeneration still needs to be unraveled.

### HUNTINGTON’S DISEASE

HD is a severe familial neurodegenerative disease typified by chorea (abnormal involuntary limb movements), incoordination, cognitive decline, and behavioral difficulties (Walker, 2007). The prevalence of this autosomal dominant heritable disease is five to seven affected individuals per 100,000 people in the Western world. HD is characterized by neuronal and glial aggregates, neuronal dysfunction and neurodegeneration, starting in the striatum and the cerebral cortex (corticostriatal pathway). An individual is affected when the polyglutamine (polyQ) repeat present in the disease-related huntingtin (HTT) protein exceed 36–41 glutamines (Walker, 2007). The encoding CAG repeats are not unique for HD as at least eight other neurodegenerative disorders are caused by a similar polyQ expansion in different proteins. Although the cause is already known for decades, the exact disease mechanism is still unclear. Interestingly, even though every cell in the body expresses the polyQ-expanded proteins, mainly neurons are affected and degenerate (Han et al., 2010). Fragments of the polyQ-expanded HTT are thought to initiate aggregation, and the resulting protein aggregates or inclusion bodies (IB) are an important hallmark of HD. More recently it became clear that these IBs are not necessarily causing neurodegeneration, as the formation of IBs did not correlate with cell death in cultured striatal neurons (Saudou et al., 1998). The formation of IBs may even be a protection mechanism of the cell to sequester toxic monomers and oligomers, thereby promoting cell survival (Arrasate et al., 2004; Truant et al., 2008).

Intriguingly, glial cells hardly show aggregates in HD patient material, while the expanded HTT is also expressed in these cells (Shin et al., 2005). Still, neuropathological studies show glial involvement in HD by phagocytosis of dead neurons and activated astrocytes (Reddy et al., 1998; Li et al., 2003), and the severity of both astrogliosis and microgliosis correlates with disease progression (Sapp et al., 2001; Faideau et al., 2010). Increased GFAP expression and activated microglia are already observed in presymptomatic stages in HD patients. With increased aggregation in neurons also the number of activated microglia and astrocytes increases (Sapp et al., 2001; Faideau et al., 2010). In addition, both microglial cell lines expressing polyQ-expanded HTT and primary microglia from early postnatal HD mice were strongly impaired in their migration toward chemotactic stimuli, and showed a retarded response to laser-induced brain injury *in vivo* (Kwan et al., 2012). This suggests that polyQ-expanded HTT expression influences the appearance and function of reactive microglia. Similarly, astrocytes showed a loss of their regulatory functions: in the R6/2 HD mouse model decreased levels of glutamate transporters and glutamate uptake were observed, eventually leading to excitotoxicity (Shin et al., 2005). Intriguingly, excitotoxicity also occurred when polyQ-expanded HTT is only expressed in striatal astrocytes but not in neurons (Faideau et al., 2010; Wang et al., 2012). In addition, astrocytes isolated from R6/2 mice suppressed the secretion of the chemokine CCL5/RANTES and of BDNF, which is a growth factor important for neuronal survival, thereby inhibiting the trophic functions of astrocytes (Chou et al., 2008). So despite the low abundance of IBs, exclusive expression of polyQ-expanded HTT in astrocytes caused a decrease in glutamate uptake followed by excitotoxicity, neurodegeneration and an age-dependent HD-like phenotype in mice (Bradford et al., 2009), suggesting a crucial role for astrocytes in HD that is directly affected by polyQ-expanded HTT. As astrocytes and microglia hardly show IB formation in HD and when they appear, they are much smaller in size (Shin et al., 2005), differences in HTT ubiquitination or proteasome activity might explain these dissimilarities.

An important role for the UPS in HD was suggested in the late nineties, when Ub and proteasomes were found to be colocalized with HTT aggregates as shown by immunohistochemistry in HD mice and post-mortem patient material (Davies et al., 1997; DiFiglia et al., 1997). This led to the hypothesis that components of the UPS were irreversibly sequestered into HTT aggregates, as was also suggested by FRAP studies that showed no fluorescence recovery after photobleaching in fluorescently tagged proteasomes that were present in aggregates (Holmberg et al., 2004). However, we recently showed with fluorescent pulse-chase experiments that proteasomes were exchanged in HTT aggregates but with slower kinetics than it would be detectable by FRAP analysis (Schipper-Krom et al., 2014). This indicates that proteasomes are dynamically recruited into HTT aggregates. Since soluble HTT can be ubiquitinated, HTT can be targeted for proteasomal degradation (Thompson et al., 2009; Juenemann et al., 2013). However, various studies have reported an impairment of the proteasome system in both HD cell models and in the brain of HD patients (Seo et al., 2004; Hunter et al., 2007). Moreover, impairment of the proteasome causes accumulation of the potentially toxic aggregation-prone N-terminal HTT fragments (Li et al., 2010). Yet, proteasomes are able to degrade polyQ-expanded HTT fragments efficiently and entirely, but only when HTT is efficiently targeted for degradation by ubiquitination (Juenemann et al., 2013). Together these results suggest a crucial role for the UPS in HD pathogenesis.

Cellular stress due to polyQ-expanded HTT in neurons can lead to proteasome subunit changes, as increased levels of the IFNγ-inducible immunoproteasome subunits LMP2 and LMP7 were observed in cortex and striatum of both HD mice and post-mortem HD patient material. Interestingly, this increase was mainly attributed to degenerating neurons (Díaz-Hernández et al., 2003), and may be the result of glial cytokine secretion. It is unknown whether glial cells upregulate immunoproteasomes in HD, and whether the induction of the immunoproteasome is beneficial in HD. While these proteasomes are better capable to deal with protein aggregates under oxidative stress (Seifert et al., 2010), it is unknown whether immunoproteasomes are also better capable to degrade polyQ-expanded HTT fragments. Still, the apparent absence of HTT aggregates in glial cells remains intriguing, and may be explained by observed differences in proteasome activity in wild type mouse brains where glial cells showed increased proteasome activity when compared to neurons. It remains, however, to be examined whether this explains the differences in aggregate size and abundance. Remarkably, when polyQ-expanded HTT was present in R6/2 mice, proteasome activity levels were not altered (Tydlacka et al., 2008), although this does not reflect any possible changes in proteasome composition. The latter finding is underscored by a recent study, where it was shown by using proteasome activity-based probes that proteasomes recruited into aggregates were accessible for substrates and remained active. Besides, no differences were observed in overlay proteasome activity between HD and wild type mice (Schipper-Krom et al., 2014). Together, this indicates that the overall activity of the proteasome does not change in HD, although the composition of proteolytic subunits undergoes some alterations. Therefore, studying the role of the UPS in glial cells in HD is of high importance.

### AMYOTROPHIC LATERAL SCLEROSIS

Amyotrophic lateral sclerosis is a late onset, rapidly progressive and ultimately fatal neurological disorder, characterized by muscle weakness and atrophy leading to the inability to control voluntary movements. These symptoms are caused by the loss of motor neurons in the brain and spinal cord. Prevalence is increasing with age from overall 4–6 cases per 100,000 to up to 33 cases per 100,000 in 60–75-year-olds. About 10% of these patients have the familial form of ALS, often caused by mutations in the gene coding for copper-zinc superoxide dismutase (SOD1; Majoor-Krakauer et al., 2003). Motor neuron degeneration is often preceded by the formation of nuclear inclusions containing ALS associated proteins SOD1, TDP-43. or FUS and are usually also Ub positive (Leigh et al., 1991; Bendotti et al., 2012). Glial pathology is strongly present in ALS as observed in cell models, mice and post-mortem patient material (Alexianu et al., 2001; Petrik et al., 2007; Evans et al., 2013). Although SOD1 expression in microglia or astrocytes alone is not sufficient to cause ALS phenotype in mice, transplantation of healthy microglia in SOD1 mutated mice slows down disease progression (Gong et al., 2000; Clement et al., 2003; Beers et al., 2006). In addition, SOD1 expression alone in motor neurons does not cause ALS pathology, suggesting that glial function is important in ALS development (Pramatarova et al., 2001). Normally, astrocytes are secreting numerous trophic factors, like VEGF and BNDF to promote neuronal survival, and they take up glutamate from the synaptic cleft. However, activated astrocytes in ALS showed disturbances in these functions, eventually leading to excitotoxicity (Trotti et al., 1999; Evans et al., 2013). Recently, it was shown that a mutation in C9orf72 could lead to aggregates in neurons, astrocytes, microglia and oligodendrocytes (Mizielinska et al., 2013). Besides, affected astrocytes caused neuronal cell death when co-cultured, suggesting an important role of astrocytes in this disease (Meyer et al., 2014). Microglia have been shown to secrete IL-1β as a response to purified mutant SOD1 stimulation, which is thus an important pro-inflammatory trigger (Meissner et al., 2010). Also secretion of several other pro-inflammatory cytokines such as TFN-α, IFNγ, and IL-6 was reported, resulting in (more) activation of astrocytes and microglia (Evans et al., 2013).

Similar to the neurodegenerative diseases described above also impairment of several components of the UPS is observed in ALS, which is likely a cause or consequence of the protein aggregates that are widely present (Kabashi et al., 2008; Bendotti et al., 2012). Impairment of the UPS but not of the autophagic pathway led to ALS-like symptoms in mice. Conditional knock-out of the 19S proteasome subunit RPT3 specifically in motor neurons caused accumulation of ALS related proteins (FUS and TDP-43) in these cells, and led to reactive gliosis and motor dysfunction. A knock-out of autophagy associated gene Atg5 only led to accumulations of Ub and p62 in the cytoplasm but not of ALS-related proteins (Tashiro et al., 2012). While impairment of the UPS in motor neurons induced an ALS-like phenotype, immunohistochemical studies showed significantly increased levels of Ub and proteasome subunits in both motor neurons and astrocytes in ALS (Aquilano et al., 2003; Mendonça et al., 2006). This contradictory result could be explained by findings in an ALS mouse model where a decrease in constitutive proteasome and an increase in immunoproteasome levels were observed, which correlated with the glia-mediated inflammatory response. Also the levels of the reactive astrocyte marker GFAP, the microglia marker CD68, and secretion of the cytokine TNF-α were upregulated in the ALS mice in a presymptomatic stage (Cheroni et al., 2009). Motor neurons in ALS also showed an upregulation of PA28αβ which could lead to a decrease in the turnover of ubiquitinated proteins as this requires the 19S activator (Bendotti et al., 2012). However, when mutant SOD1 mice were crossed with β1i/LMP2 knockout mice, the absence of immunoproteasomes *in vivo* could not significantly prevent mutant SOD1-induced disease; as no changes in disease symptoms were observed (Puttaparthi et al., 2007). So while activated astrocytes and microglia secrete cytokines that result in the induction of immunoproteasomes in both glia and neurons, these changes seem to be not beneficial in the clearance of accumulating SOD1 proteins.

## CONCLUDING REMARKS AND FUTURE PERSPECTIVES

Studies on the role of the UPS in neurodegenerative disease are mainly focusing on neurons, as these diseases are often considered to be cell autonomous due to the fact that neurons appear to be the most severe affected. Recently, however, the role of glial cells in neurodegenerative diseases is emerging. The fact that glial activation can already be observed in early presymptomatic stages advocates for an important if not crucial role for glial cells in the initiation of these diseases (Tai et al., 2007; Faideau et al., 2010). Glial cells express most of the disease-related proteins, and glial aggregates are observed in some of these neurodegenerative diseases albeit with lower frequencies (Bruijn et al., 1997; Shin et al., 2005; Tong et al., 2014). In addition, the disease-causing proteins can alter glial function by the induction of secretion of various cytokines. This makes glial cells important players in the various diseases, as the interplay between disease-causing proteins and the altered UPS not only affects glial cell homeostasis, but indirectly also neuronal function. A common hallmark of the here discussed various neurodegenerative diseases is the general downregulation of the constitutive proteasome accompanied by an upregulated immunoproteasome. This is in line with the observed phenotypical alterations that microglia and astrocytes undergo during disease progression, and the resulting pro-inflammatory environment correlates perfectly with the induction of the immunoproteasome. The induced immunoproteasomes may be better able to degrade intracellular protein aggregates, as suggested for protein aggregates that are induced following IFNγ-induced stress (Seifert et al., 2010). Since IFNγ is secreted by microglia (Kawanokuchi et al., 2006) and is able to activate both microglia and astrocytes in several neurodegenerative diseases (Choi et al., 2013; Evans et al., 2013), most likely the observed immunoproteasome induction is mediated by microglia. Nevertheless, while immunoproteasomes are directly induced by IFNγ, this cytokine also induces the accumulation of oxidized proteins that may accelerate aggregation and affect protein homeostasis in time. Still, the induced immunoproteasomes appear better capable to degrade polyubiquitinated proteins under these circumstances, and proteolytic activity of immunoproteasomes is higher compared to standard proteasomes (Seifert et al., 2010). These data suggest that activation of glia and induction of the immunoproteasome may be beneficial for clearing disease-causing proteins.

Yet, similar to chronic activation of glia, it is unknown whether induction of the immunoproteasome is on the long term beneficial or detrimental in neurodegenerative diseases. After all, the increased immunoproteasome induction in these diseases and in aging could also indicate undesirable protein accumulation and chronic inflammation (Díaz-Hernández et al., 2003; Mishto et al., 2006; Cheroni et al., 2009; Nijholt et al., 2011; Orre et al., 2013; Bellavista et al., 2014). When the induction of the immunoproteasome is not beneficial in targeting the accumulating proteins, specific inhibitors targeting immunoproteasome subunits could be an efficient therapeutic approach. Although several of these inhibitors are already developed (Miller et al., 2013), more insight is required in the exact function of the immunoproteasome in these disorders.

Next to focusing on proteasome activity, for future approaches it is also important to examine which other alterations in the UPS observed in glia can be used as possible therapeutic targets. Although proteasome compositional changes appear in glial cells during disease progression, in general proteasomes stay active and accessible even in cells showing large protein aggregates (Tydlacka et al., 2008; Basler et al., 2013; Orre et al., 2013; Schipper-Krom et al., 2014). Yet, it is unclear whether the diseased proteins are correctly targeted for degradation, as inefficient ubiquitination of mutant and misfolded proteins could explain the observed protein accumulations and neuronal vulnerability. Improved targeting of the disease-causing proteins toward the proteasome by modifying ubiquitination (or with other Ub-like proteins) would increase their degradation and delay onset of disease. It is unknown whether the ubiquitination patterns of the various disease-related proteins in glia and neurons is dissimilar and whether they change during disease progression. Therefore, determining the ubiquitination patterns of these proteins in different stages of disease in both neurons and glia, along with determining the involved specific ubiquitin ligases and DUBs in these cells should lead to the identification of new and more specific therapeutic targets.

## Conflict of Interest Statement

The authors declare that the research was conducted in the absence of any commercial or financial relationships that could be construed as a potential conflict of interest.

## References

[B1] Abbott N. J. (2002). Astrocyte–endothelial interactions and blood–brain barrier permeability. *J. Anat.* 200 629–638 10.1046/j.1469-7580.2002.00064.x 12162730 PMC1570746

[B2] Alexianu M. E. Kozovska M. Appel S. H. (2001). Immune reactivity in a mouse model of familial ALS correlates with disease progression. *Neurology* 57 1282–1289 10.1212/WNL.57.7.1282 11591849

[B3] Ali S. F. Binienda Z. K. Imam S. Z. (2011). Molecular aspects of dopaminergic neurodegeneration: gene–environment interaction in Parkin dysfunction. *Int. J. Environ. Res. Public Health* 8 4702–4713 10.3390/ijerph8124702 22408597 PMC3290988

[B4] Alliot F. Godin I. Pessac B. (1999). Microglia derive from progenitors, originating from the yolk sac, and which proliferate in the brain. *Dev. Brain Res.* 117 145–152 10.1016/S0165-3806(99)00113-3 10567732

[B5] Aloisi F. (2001). Immune function of microglia. *Glia* 36 165–179 10.1002/glia.1106 11596125

[B6] Amor S. Peferoen L. A. Vogel D. Y. Breur M. Van Der Valk P. Baker D. (2013). Inflammation in neurodegenerative diseases – an update. *Immunology* 142 151–166 10.1111/imm.12233 24329535 PMC4008224

[B7] Aquilano K. Rotilio G. Ciriolo M. R. (2003). Proteasome activation and nNOS down-regulation in neuroblastoma cells expressing a Cu, Zn superoxide dismutase mutant involved in familial ALS. *J. Neurochem.* 85 1324–1335 10.1046/j.1471-4159.2003.01783.x 12753090

[B8] Arrasate M. Mitra S. Schweitzer E. S. Segal M. R. Finkbeiner S. (2004). Inclusion body formation reduces levels of mutant huntingtin and the risk of neuronal death. *Nature* 431 805–810 10.1038/nature02998 15483602

[B9] Basler M. Kirk C. J. Groettrup M. (2013). The immunoproteasome in antigen processing and other immunological functions. *Curr. Opin. Immunol.* 25 74–80 10.1016/j.coi.2012.11.004 23219269

[B10] Baumann N. Pham-Dinh D. (2001). Biology of oligodendrocyte and myelin in the mammalian central nervous system. *Physiol. Rev.* 81 871–927 11274346 10.1152/physrev.2001.81.2.871

[B11] Baumeister W. Walz J. Zühl F. Seemüller E. (1998). The proteasome: paradigm of a self-compartmentalizing protease. *Cell* 92 367–380 10.1016/S0092-8674(00)80929-0 9476896

[B12] Bedford L. Hay D. Devoy A. Paine S. Powe D. G. Seth R. (2008). Depletion of 26S proteasomes in mouse brain neurons causes neurodegeneration and Lewy-like inclusions resembling human pale bodies. *J. Neurosci.* 28 8189–8198 10.1523/JNEUROSCI.2218-08.2008 18701681 PMC6670564

[B13] Beers D. R. Henkel J. S. Xiao Q. Zhao W. Wang J. Yen A. A. (2006). Wild-type microglia extend survival in PU.1 knockout mice with familial amyotrophic lateral sclerosis *Proc. Nati. Acad. Sci. U.S.A.* 103 16021–16026 10.1073/pnas.0607423103 PMC1613228 17043238

[B14] Bellavista E. Santoro A. Galimberti D. Comi C. Luciani F. Mishto M. (2014). Current understanding on the role of standard and immunoproteasomes in inflammatory/immunological pathways of multiple sclerosis. *Autoimmune Dis.* 2014 739705 10.1155/2014/739705 PMC3910067 24523959

[B15] Bendotti C. Marino M. Cheroni C. Fontana E. Crippa V. Poletti A. (2012). Dysfunction of constitutive and inducible ubiquitin–proteasome system in amyotrophic lateral sclerosis: implication for protein aggregation and immune response. *Prog. Neurobiol.* 97 101–126 10.1016/j.pneurobio.2011.10.001 22033150

[B16] Bi W. Jing X. Zhu L. Liang Y. Liu J. Yang L. (2012). Inhibition of 26S protease regulatory subunit 7 (MSS1) suppresses neuroinflammation. *PLoS ONE* 7:e36142 10.1371/journal.pone.0036142 PMC3356363 22629310

[B17] Bozzali M. Padovani A. Caltagirone C. Borroni B. (2011). Regional grey matter loss and brain disconnection across Alzheimer disease evolution. *Curr. Med. Chem.* 18 2452–2458 10.2174/092986711795843263 21568913

[B18] Bradford J. Shin J. Roberts M. Wang C. Li X. Li S. (2009). Expression of mutant huntingtin in mouse brain astrocytes causes age-dependent neurological symptoms. *Proc. Natl. Acad. Sci. U.S.A.* 106 22480–22485 10.1073/pnas.0911503106 20018729 PMC2799722

[B19] Bradl M. Lassmann H. (2010). Oligodendrocytes: biology and pathology. *Acta Neuropathol.* 119 37–53 10.1007/s00401-009-0601-5 19847447 PMC2799635

[B20] Brenner M. Johnson A. B. Boespflug-Tanguy O. Rodriguez D. Goldman J. E. Messing A. (2001). Mutations in Gfap, encoding glial fibrillary acidic protein, are associated with Alexander disease. *Nat. Genet.* 27 117–120 10.1038/83679 11138011

[B21] Brettschneider J. Del Tredici K. Toledo J. B. Robinson J. L. Irwin D. J. Grossman M. (2013). Stages of pTDP-43 pathology in amyotrophic lateral sclerosis. *Ann. Neurol.* 74 20–38 10.1002/ana.23937 23686809 PMC3785076

[B22] Bruijn L. I. Becher M. W. Lee M. K. Anderson K. L. Jenkins N. A. Copeland N. G. (1997). ALS-linked SOD1 mutant G85R mediates damage to astrocytes and promotes rapidly progressive disease with SOD1-containing inclusions. *Neuron* 18 327–338 10.1016/S0896-6273(00)80272-X 9052802

[B23] Casarejos M. J. Solano R. M. Rodriguez-Navarro J. A. Gómez A. Perucho J. Castaño J. G. (2009). Parkin deficiency increases the resistance of midbrain neurons and glia to mild proteasome inhibition: the role of autophagy and glutathione homeostasis. *J. Neurochem.* 110 1523–1537 10.1111/j.1471-4159.2009.06248.x 19549073

[B24] Cheroni C. Marino M. Tortarolo M. Veglianese P. De Biasi S. Fontana E. (2009). Functional alterations of the ubiquitin-proteasome system in motor neurons of a mouse model of familial amyotrophic lateral sclerosis. *Hum. Mol. Genet.* 18 82–96 10.1093/hmg/ddn319 18826962 PMC3298865

[B25] Cho W. Messing A. (2009). Properties of astrocytes cultured from GFAP over-expressing and GFAP mutant mice. *Exp. Cell Res.* 315 1260–1272 10.1016/j.yexcr.2008.12.012 19146851 PMC2665202

[B26] Choi K. Park J. Lee J. Han E. C. Choi C. (2013). Mutant ubiquitin attenuates interleukin-1beta- and tumor necrosis factor-alpha-induced pro-inflammatory signaling in human astrocytic cells. *PLoS ONE* 8:e67891 10.1371/journal.pone.0067891 PMC3700915 23844119

[B27] Chou S. Y. Weng J. Y. Lai H. L. Liao F. Sun S. H. Tu P. H. (2008). Expanded-polyglutamine huntingtin protein suppresses the secretion and production of a chemokine (CCL5/RANTES) by astrocytes. *J. Neurosci.* 28 3277–3290 10.1523/JNEUROSCI.0116-08.2008 18367595 PMC6670608

[B28] Chu C. T. Caruso J. L. Cummings T. J. Ervin J. Rosenberg C. Hulette C. M. (2000). Ubiquitin immunochemistry as a diagnostic aid for community pathologists evaluating patients who have dementia. *Mod. Pathol.* 13 420–426 10.1038/modpathol.3880072 10786809

[B29] Chung W. S. Clarke L. E. Wang G. X. Stafford B. K. Sher A. Chakraborty C. (2013). Astrocytes mediate synapse elimination through MEGF10 and MERTK pathways. *Nature* 504 394–400 10.1038/nature12776 24270812 PMC3969024

[B30] Clement A. M. Nguyen M. D. Roberts E. A. Garcia M. L. Boillée S. Rule M. (2003). Wild-type nonneuronal cells extend survival of SOD1 mutant motor neurons in ALS mice. *Science* 302 113–117 10.1126/science.1086071 14526083

[B31] Cumiskey D. O’Connor J. J. Pickering M. (2005). Actions of TNF-alpha on glutamatergic synaptic transmission in the central nervous system. *Exp. Physiol.* 90 663–670 10.1113/expphysiol.2005.030734 15944202

[B32] Dalal N. V. Pranski E. L. Tansey M. G. Lah J. J. Levey A. I. Betarbet R. S. (2012). RNF11 modulates microglia activation through NF-κB signalling cascade. *Neurosci. Lett.* 528 174–179 10.1016/j.neulet.2012.08.060 22975135 PMC3478679

[B33] Davies S. W. Turmaine M. Cozens B. A. Difiglia M. Sharp A. H. Ross C. A. (1997). Formation of neuronal intranuclear inclusions underlies the neurological dysfunction in mice transgenic for the HD mutation. *Cell* 90 537–548 10.1016/S0092-8674(00)80513-9 9267033

[B34] de Lau L. M. Breteler M. M. (2006). Epidemiology of Parkinson’s disease. *Lancet Neurol.* 5 525–535 10.1016/S1474-4422(06)70471-9 16713924

[B35] Díaz-Hernández M. Hernández F. Martín-Aparicio E. Gómez-Ramos P. Morán M. A. Castaño J. G. (2003). Neuronal induction of the immunoproteasome in Huntington’s disease. *J. Neurosci.* 23 11653–11661 14684867 10.1523/JNEUROSCI.23-37-11653.2003 PMC6740941

[B36] DiFiglia M. Sapp E. Chase K. O. Davies S. W. Bates G. P. Vonsattel J. P. (1997). Aggregation of huntingtin in neuronal intranuclear inclusions and dystrophic neurites in brain. *Science* 277 1990–1993 10.1126/science.277.5334.1990 9302293

[B37] Ding Q. Dimayuga E. Markesbery W. R. Keller J. N. (2004). Proteasome inhibition increases DNA and RNA oxidation in astrocyte and neuron cultures. *J. Neurochem.* 91 1211–1218 10.1111/j.1471-4159.2004.02802.x 15569264

[B38] Elkharaz J. Ugun-Klusek A. Constantin-Teodosiu T. Lawler K. Mayer R. J. Billett E. (2013). Implications for oxidative stress and astrocytes following 26S proteasomal depletion in mouse forebrain neurones. *Biochim. Biophys. Acta* 1832 1930–1938 10.1016/j.bbadis.2013.07.002 23851049

[B39] Evans M. C. Couch Y. Sibson N. Turner M. R. (2013). Inflammation and neurovascular changes in amyotrophic lateral sclerosis. *Mol. Cell. Neurosci.* 53 34–41 10.1016/j.mcn.2012.10.008 23110760

[B40] Faideau M. Kim J. Cormier K. Gilmore R. Welch M. Auregan G. (2010). *In vivo* expression of polyglutamine-expanded huntingtin by mouse striatal astrocytes impairs glutamate transport: a correlation with Huntington’s disease subjects. *Hum. Mol. Genet.* 19 3053–3067 10.1093/hmg/ddq212 20494921 PMC2901144

[B41] Ferrer I. Lopez-Gonzalez I. Carmona M. Arregui L. Dalfo E. Torrejon-Escribano B. (2014). Glial and neuronal tau pathology in tauopathies: characterization of disease-specific phenotypes and tau pathology progression. *J. Neuropathol. Exp. Neurol.* 73 81–97 10.1097/NEN.0000000000000030 24335532

[B42] Forsberg K. Andersen P. Marklund S. L. Brannstrom T. (2011). Glial nuclear aggregates of superoxide dismutase-1 are regularly present in patients with amyotrophic lateral sclerosis. *Acta Neuropathol.* 121 623–634 10.1007/s00401-011-0805-3 21287393 PMC3085063

[B43] Ginhoux F. Greter M. Leboeuf M. Nandi S. See P. Gokhan S. (2010). Fate mapping analysis reveals that adult microglia derive from primitive macrophages. *Science* 330 841–845 10.1126/science.1194637 20966214 PMC3719181

[B44] Glickman M. H. Ciechanover A. (2002). The ubiquitin–proteasome proteolytic pathway: destruction for the sake of construction. *Physiol. Rev.* 82 373–428 10.1152/physrev.00027.2001 11917093

[B45] Goldbaum O. Riedel M. Stahnke T. Richter-Landsberg C. (2009). The small heat shock protein HSP25 protects astrocytes against stress induced by proteasomal inhibition. *Glia* 57 1566–1577 10.1002/glia.20870 19330846

[B46] Goldbaum O. Vollmer G. Richter-Landsberg C. (2006). Proteasome inhibition by MG-132 induces apoptotic cell death and mitochondrial dysfunction in cultured rat brain oligodendrocytes but not in astrocytes. *Glia* 53 891–901 10.1002/glia.20348 16609961

[B47] Gong Y. H. Parsadanian A. S. Andreeva A. Snider W. D. Elliott J. L. (2000). Restricted expression of G86R Cu/Zn superoxide dismutase in astrocytes results in astrocytosis but does not cause motoneuron degeneration. *J. Neurosci.* 20 660–665 10632595 10.1523/JNEUROSCI.20-02-00660.2000 PMC6772423

[B48] Gray F. Scaravilli F. Everall I. Chretien F. An S. Boche D. (1996). Neuropathology of early HIV-1 infection. *Brain Pathol.* 6 1–15 10.1111/j.1750-3639.1996.tb00775.x 8866743

[B49] Gregori L. Fuchs C. Figueiredo-Pereira M. E. Van Nostrand W. E. Goldgaber D. (1995). Amyloid β-protein inhibits ubiquitin-dependent protein degradation *in vitro*. *J. Biol. Chem.* 270 19702–19708 10.1074/jbc.270.34.19702 7649980

[B50] Griffin W. S. Stanley L. C. Ling C. White L. Macleod V. Perrot L. J. (1989). Brain interleukin 1 and S-100 immunoreactivity are elevated in Down syndrome and Alzheimer disease. *Proc. Natl. Acad. Sci. U.S.A.* 86 7611–7615 10.1073/pnas.86.19.7611 2529544 PMC298116

[B51] Gu X. L. Long C. X. Sun L. Xie C. Lin X. Cai H. (2010). Astrocytic expression of Parkinson’s disease-related A53T alpha-synuclein causes neurodegeneration in mice. *Mol. Brain* 3 12 10.1186/1756-6606-3-12 PMC2873589 20409326

[B52] Halliday G. M. Stevens C. H. (2011). Glia: initiators and progressors of pathology in Parkinson’s disease. *Mov. Disord.* 26 6–17 10.1002/mds.23455 21322014

[B53] Han I. You Y. Kordower J. H. Brady S. T. Morfini G. A. (2010). Differential vulnerability of neurons in Huntington’s disease: the role of cell type-specific features. *J. Neurochem.* 113 1073–1091 10.1111/j.1471-4159.2010.06672.x 20236390 PMC2890032

[B54] Hardy J. Lewis P. Revesz T. Lees A. Paisan-Ruiz C. (2009). The genetics of Parkinson’s syndromes: a critical review. *Curr. Opin. Genet. Dev.* 19 254–265 10.1016/j.gde.2009.03.008 19419854

[B55] Hipp M. S. Kalveram B. Raasi S. Groettrup M. Schmidtke G. (2005). FAT10 a ubiquitin-independent signal for proteasomal degradation. *Mol. Cell. Biol.* 25 3483–3491 10.1128/MCB.25.9.3483-3491.2005 15831455 PMC1084302

[B56] Holmberg C. I. Staniszewski K. E. Mensah K. N. Matouschek A. Morimoto R. I. (2004). Inefficient degradation of truncated polyglutamine proteins by the proteasome. *EMBO J.* 23 4307–4318 10.1038/sj.emboj.7600426 15470501 PMC524390

[B57] Hunter J. M. Lesort M. Johnson G. V. W. (2007). Ubiquitin-proteasome system alterations in a striatal cell model of Huntington’s disease. *J. Neurosci. Res.* 85 1774–1788 10.1002/jnr.21287 17455294

[B58] Imai Y. Soda M. Takahashi R. (2000). Parkin suppresses unfolded protein stress-induced cell death through its E3 ubiquitin–protein ligase activity. *J. Biol. Chem.* 275 435661–435664 10.1074/jbc.C000447200 10973942

[B59] Juenemann K. Schipper-Krom S. Wiemhoefer A. Kloss A. Sanz Sanz A. Reits E. A. J. (2013). Expanded polyglutamine-containing N-terminal huntingtin fragments are entirely degraded by mammalian proteasomes. *J. Biol. Chem.* 288 27068–27084 10.1074/jbc.M113.486076 23908352 PMC3779707

[B60] Kabashi E. Agar J. N. Hong Y. Taylor D. M. Minotti S. Figlewicz D. A. (2008). Proteasomes remain intact, but show early focal alteration in their composition in a mouse model of amyotrophic lateral sclerosis. *J. Neurochem.* 105 2353–2366 10.1111/j.1471-4159.2008.05317.x 18315558

[B61] Kamphuis W. Orre M. Kooijman L. Dahmen M. Hol E. M. (2012). Differential cell proliferation in the cortex of the APPswePS1dE9 Alzheimer’s disease mouse model. *Glia* 60 615–629 10.1002/glia.22295 22262260

[B62] Kawanokuchi J. Mizuno T. Takeuchi H. Kato H. Wang J. Mitsuma N. (2006). Production of interferon-gamma by microglia. *Mult. Scler.* 12 558–564 10.1177/1352458506070763 17086900

[B63] Keller J. N. Hanni K. B. Markesbery W. R. (2000). Impaired proteasome function in Alzheimer’s Disease. *J. Neurochem.* 75 436–439 10.1046/j.1471-4159.2000.0750436.x 10854289

[B64] Kettenmann H. Kirchhoff F. Verkhratsky A. (2013). Microglia: new roles for the synaptic stripper. *Neuron* 77 10–18 10.1016/j.neuron.2012.12.023 23312512

[B65] Kettenmann H. Verkhratsky A. (2008). Neuroglia: the 150 years after. *Trends Neurosci.* 31 653–659 10.1016/j.tins.2008.09.003 18945498

[B66] Kraft A. W. Hu X. Yoon H. Yan P. Xiao Q. Wang Y. (2013). Attenuating astrocyte activation accelerates plaque pathogenesis in APP/PS1 mice. *FASEB J.* 27 187–198 10.1096/fj.12-208660 23038755 PMC3528309

[B67] Kuzdas D. Stemberger S. Gaburro S. Stefanova N. Singewald N. Wenning G. K. (2013). Oligodendroglial alpha-synucleinopathy and MSA-like cardiovascular autonomic failure: experimental evidence. *Exp. Neurol.* 247 531–536 10.1016/j.expneurol.2013.02.002 23399889 PMC3748345

[B68] Kwan W. Trager U. Davalos D. Chou A. Bouchard J. Andre R. (2012). Mutant huntingtin impairs immune cell migration in Huntington disease. *J. Clin. Invest.* 122 4737–4747 10.1172/JCI64484 23160193 PMC3533551

[B69] Kwon S. Ahn T. Yoon M. Jeon B. S. (2008). Bv-2 stimulation by lactacystin results in a strong inflammatory reaction and apoptotic neuronal death in SH-SY5Y cells. *Brain Res.* 1205 116–121 10.1016/j.brainres.2008.02.030 18353281

[B70] Lam Y. A. Pickart C. M. Alban A. Landon M. Jamieson C. Ramage R. (2000). Inhibition of the ubiquitin-proteasome system in Alzheimer’s disease. *Proc. Natl. Acad. Sci. U.S.A.* 97 9902–9906 10.1073/pnas.170173897 10944193 PMC27620

[B71] Lee H. Suk J. Patrick C. Bae E. Cho J. Rho S. (2010). Direct transfer of a-synuclein from neuron to astroglia causes inflammatory responses in synucleinopathies. *J. Biol. Chem.* 285 9262–9272 10.1074/jbc.M109.081125 20071342 PMC2838344

[B72] Leigh P. N. Whitwell H. Garofalo O. Buller J. Swash M. Martin J. E. (1991). Ubiquitin-immunoreactive intraneuronal inclusions in amyotrophic lateral sclerosis. Morphology, distribution, and specificity *Brain J. Neurol.* 114 775–788 10.1093/brain/114.2.775 1646064

[B73] Li S. Yu Z. Li C. Nguyen H. Zhou Y. Deng C. (2003). Lack of huntingtin-associated protein-1 causes neuronal death resembling hypothalamic degeneration in Huntington’s disease. *J. Neurosci.* 23 6956–6964 12890790 10.1523/JNEUROSCI.23-17-06956.2003 PMC6740731

[B74] Li X. Wang C. Huang S. Xu X. Li X. Li H. (2010). Inhibiting the ubiquitin–proteasome system leads to preferential accumulation of toxic N-terminal mutant huntingtin fragments. *Hum. Mol. Genet.* 19 2445–2455 10.1093/hmg/ddq127 20354076 PMC2876889

[B75] Lilienbaum A. (2013). Relationship between the proteasomal system and autophagy. *Int. J. Biochem. Mol. Biol.* 4 1–26 23638318 PMC3627065

[B76] Lin Z. Zhao D. Yang L. (2013). Interaction between misfolded PrP and the ubiquitin-proteasome system in prion-mediated neurodegeneration. *Acta Biochim. Biophys. Sin.* 45 477–484 10.1093/abbs/gmt020 23449072

[B77] Lisak R. P. Nedelkoska L. Studzinski D. Bealmear B. Xu W. Benjamins J. A. (2011). Cytokines regulate neuronal gene expression: differential effects of Th1 Th2 and monocyte/macrophage cytokines. *J. Neuroimmunol.* 238 19–33 10.1016/j.jneuroim.2011.06.010 21803433

[B78] Lopez Salon M. Pasquini L. Besio Moreno M. Pasquini J. M. Soto E. (2003). Relationship between β-amyloid degradation and the 26S proteasome in neural cells. *Exp. Neurol.* 180 131–143 10.1016/S0014-4886(02)00060-2 12684027

[B79] Lowe J. Blanchard A. Morrell K. Lennox G. Reynolds L. Billett M. (1988). Ubiquitin is a common factor in intermediate filament inclusion bodies of diverse type in man, including those of Parkinson’s disease, Pick’s disease, and Alzheimer’s disease, as well as Rosenthal fibres in cerebellar astrocytomas, cytoplasmic bodies in muscle, and mallory bodies in alcoholic liver disease. *J. Pathol.* 155 9–15 10.1002/path.1711550105 2837558

[B80] Majoor-Krakauer D. Willems P. Hofman A. (2003). Genetic epidemiology of amyotrophic lateral sclerosis. *Clin. Genet.* 63 83–101 10.1046/j.0009-9163.2002.00001.x 12630951

[B81] Mamber C. Kozareva D. A. Kamphuis W. Hol E. M. (2013). Shades of gray: the delineation of marker expression within the adult rodent subventricular zone. *Prog. Neurobiol.* 111 1–16 10.1016/j.pneurobio.2013.07.003 23994259

[B82] Mandrekar S. Jiang Q. Lee C. Y. Koenigsknecht-Talboo J. Holtzman D. M. Landreth G. E. (2009). Microglia mediate the clearance of soluble Abeta through fluid phase macropinocytosis. *J. Neurosci.* 29 4252–4262 10.1523/JNEUROSCI.5572-08.2009 19339619 PMC3034143

[B83] Mariani E. Polidori M. C. Cherubini A. Mecocci P. (2005). Oxidative stress in brain aging, neurodegenerative and vascular diseases: an overview. *J. Chromatogr.* 827 65–75 10.1016/j.jchromb.2005.04.023 16183338

[B84] McNaught K. S. Belizaire R. Isacson O. Jenner P. Olanow C. W. (2003). Altered proteasomal function in sporadic Parkinson’s disease. *Exp. Neurol.* 179 38–46 10.1006/exnr.2002.8050 12504866

[B85] Meissner F. Molawi K. Zychlinsky A. (2010). Mutant superoxide dismutase 1-induced IL-1ß accelerates Als pathogenesis. *Proc. Natl. Acad. Sci. U.S.A.* 107 13046–13050 10.1073/pnas.1002396107 20616033 PMC2919927

[B86] Mendonça D. M. F. Chimelli L. Martinez A. M. B. (2006). Expression of ubiquitin and proteasome in motorneurons and astrocytes of spinal cords from patients with amyotrophic lateral sclerosis. *Neurosci. Lett.* 404 315–319 10.1016/j.neulet.2006.06.009 16806703

[B87] Meyer K. Ferraiuolo L. Miranda C. J. Likhite S. Mcelroy S. Renusch S. (2014). Direct conversion of patient fibroblasts demonstrates non-cell autonomous toxicity of astrocytes to motor neurons in familial and sporadic ALS. *Proc. Natl. Acad. Sci. U.S.A.* 111 829–832 10.1073/pnas.1314085111 24379375 PMC3896192

[B88] Middeldorp J. Kamphuis W. Sluijs J. A. Achoui D. Leenaars C. H. C. Feenstra M. G. (2009). Intermediate filament transcription in astrocytes is repressed by proteasome inhibition. *FASEB J.* 23 2710–2726 10.1096/fj.08-127696 19332645 PMC3221645

[B89] Miller Z. Ao L. Kim K. B. Lee W. (2013). Inhibitors of the immunoproteasome: current status and future directions. *Curr. Pharm. Des.* 19 4140–4151 10.2174/1381612811319220018 23181576 PMC3821965

[B90] Mirza B. Hadberg H. Thomsen P. Moos T. (1999). The absence of reactive astrocytosis is indicative of a unique inflammatory process in Parkinson’s disease. *Neuroscience* 95 425–432 10.1016/S0306-4522(99)00455-8 10658622

[B91] Mishto M. Bellavista E. Ligorio C. Textoris-Taube K. Santoro A. Giordano M. (2010). Immunoproteasome LMP2 60HH variant alters MBP epitope generation and reduces the risk to develop multiple sclerosis in Italian female population. *PLoS ONE* 5:e9287 10.1371/journal.pone.0009287 PMC2823778 20174631

[B92] Mishto M. Bellavista E. Santoro A. Stolzing A. Ligorio C. Nacmias B. (2006). Immunoproteasome and LMP2 polymorphism in aged and Alzheimer’s disease brains. *Neurobiol. Aging* 27 54–66 10.1016/j.neurobiolaging.2004.12.004 16298241

[B93] Mizielinska S. Lashley T. Norona F. E. Clayton E. L. Ridler C. E. Fratta P. (2013). C9orf72 frontotemporal lobar degeneration is characterised by frequent neuronal sense and antisense RNA foci. *Acta Neuropathol.* 126 845–857 10.1007/s00401-013-1200-z 24170096 PMC3830745

[B94] Molofsky A. V. Krenick R. Ullian E. Tsai H. Deneen B. Richardson W. D. (2012). Astrocytes and disease: a neurodevelopmental perspective. *Genes Dev.* 26 891–907 10.1101/gad.188326.112 22549954 PMC3347787

[B95] Mori F. Tanji K. Odagiri S. Hattori M. Hoshikawa Y. Kono C. (2012). Ubiquitin-related proteins in neuronal and glial intranuclear inclusions in intranuclear inclusion body disease. *Pathol. Int.* 62 407–411 10.1111/j.1440-1827.2012.02812.x 22612509

[B96] Mrak R. E. (2009). Neuropathology and the neuroinflammation idea. *J. Alzheimers Dis.* 18 473–481 10.3233/JAD-2009-1158 19584454

[B97] Mulder S. D. Nielsen H. M. Blankenstein M. A. Eikelenboom P. Veerhuis R. (2014). Apolipoproteins E and J interfere with amyloid-beta uptake by primary human astrocytes and microglia *in vitro*. Glia 62 493–503 10.1002/glia.22619 24446231

[B98] Nijholt D. A. De Graaf T. R. Van Haastert E. S. Oliveira A. O. Berkers C. R. Zwart R. (2011). Endoplasmic reticulum stress activates autophagy but not the proteasome in neuronal cells: implications for Alzheimer’s disease. *Cell Death. Differ.* 18 1071–1081 10.1038/cdd.2010.176 21252911 PMC3131935

[B99] Orre M. Kamphuis W. Dooves S. Kooijman L. Chan E. T. Kirk C. J. (2013). Reactive glia show increased immunoproteasome activity in Alzheimer’s disease. *Brain* 136 1415–1431 10.1093/brain/awt083 23604491

[B100] Pappalardo L. W. Samad O. A. Black J. A. Waxman S. G. (2014). Voltage-gated sodium channel Na 1.5 contributes to astrogliosis in an *in vitro* model of glial injury via reverse Na/Ca exchange. *Glia* 62 1162–1175 10.1002/glia.22671 24740847 PMC4060891

[B101] Parpura V. Heneka M. T. Montana V. Oliet S. H. R. Schousboe A. Haydon P. G. (2012). Glial cells in (patho)physiology. *J. Neurochem.* 121 4–27 10.1111/j.1471-4159.2012.07664.x 22251135 PMC3304021

[B102] Pasanen P. Myllykangas L. Siitonen M. Raunio A. Kaakkola S. Lyytinen J. (2014). A novel α-synuclein mutation A53E associated with atypical multiple system atrophy and Parkinson’s disease-type pathology. *Neurobiol. Aging* 35 2180 10.1016/j.neurobiolaging.2014.03.024 24746362

[B103] Pekny M. Wilhelmsson U. Pekna M. (2014). The dual role of astrocyte activation and reactive gliosis. *Neurosci. Lett.* 565 30 10.1016/j.neulet.2013.12.071 24406153

[B104] Pelvig D. P. Pakkenberg H. Stark A. K. Pakkenberg B. (2008). Neocortical glial cell numbers in human brains. *Neurobiol. Aging* 29 1754–1762 10.1016/j.neurobiolaging.2007.04.013 17544173

[B105] Petrik M. S. Wilson J. M. Grant S. C. Blackband S. J. Tabata R. C. Shan X. (2007). Magnetic resonance microscopy and immunohistochemistry of the CNS of the mutant Sod murine model of ALS reveals widespread neural deficits. *Neuromolecular Med.* 9 216–229 10.1007/s12017-007-8002-1 17914180

[B106] Pintado C. Gavilan M. P. Gavilan E. Garcia-Cuervo L. Gutierrez A. Vitorica J. (2012). Lipopolysaccharide-induced neuroinflammation leads to the accumulation of ubiquitinated proteins and increases susceptibility to neurodegeneration induced by proteasome inhibition in rat hippocampus. *J. Neuroinflammation* 9 87 10.1186/1742-2094-9-87 PMC3462674 22559833

[B107] Popescu B. F. Pirko I. Lucchinetti C. F. (2013). Pathology of multiple sclerosis: where do we stand? *Continuum (Minneapolis, Minn.)* 19 901–921 10.1212/01.CON.0000433291.23091.65 PMC3915566 23917093

[B108] Pramatarova A. Laganiere J. Roussel J. Brisebois K. Rouleau G. A. (2001). Neuron-specific expression of mutant superoxide dismutase 1 in transgenic mice does not lead to motor impairment. *J. Neurosci.* 21 3369–3374 11331366 10.1523/JNEUROSCI.21-10-03369.2001 PMC6762496

[B109] Puttaparthi K. Van Kaer L. Elliott J. L. (2007). Assessing the role of immuno-proteasomes in a mouse model of familial ALS. *Exp. Neurol.* 206 53–58 10.1016/j.expneurol.2007.03.024 17482163 PMC2692686

[B110] Realini C. Dubiel W. Pratt G. Ferrell K. Rechsteiner M. (1994). Molecular cloning and expression of a gamma-interferon-inducible activator of the multicatalytic protease. *J. Biol. Chem.* 269 20727–20732 8051173

[B111] Rechsteiner M. Hill C. P. (2005). Mobilizing the proteolytic machine: cell biological roles of proteasome activators and inhibitors. *Trends Cell Biol.* 15 27–33 10.1016/j.tcb.2004.11.003 15653075

[B112] Reddy P. H. Williams M. Charles V. Garrett L. Pike-Buchanan L. Whetsell W. O. (1998). Behavioural abnormalities and selective neuronal loss in HD transgenic mice expressing mutated full-length HD cDNA. *Nat. Genet.* 20 198–202 10.1038/2510 9771716

[B113] Rock K. L. Gramm C. Rothstein L. Clark K. Stein R. Dick L. (1994). Inhibitors of the proteasome block the degradation of most cell proteins and the generation of peptides presented on MHC class I molecules. *Cell* 78 761–771 10.1016/S0092-8674(94)90462-6 8087844

[B114] Rozemuller J. M. Eikelenboom P. Stam F. C. (1986). Role of microglia in plaque formation in senile dementia of the Alzheimer type. An immunohistochemical study. *Virchows Arch. B Cell Pathol. Incl. Mol. Pathol.* 51 247–254 10.1007/BF02899034 2874657

[B115] Sapp E. Kegel K. B. Aronin N. Hashikawa T. Uchiyama Y. Tohyama K. (2001). Early and progressive accumulation of reactive microglia in the Huntington disease brain. *J. Neuropathol. Exp. Neurol.* 60 161–172 11273004 10.1093/jnen/60.2.161

[B116] Saudou F. Finkbeiner S. Devys D. Greenberg M. E. (1998). Huntingtin acts in the nucleus to induce apoptosis but death does not correlate with the formation of intranuclear inclusions. *Cell* 95 55–66 10.1016/S0092-8674(00)81782-1 9778247

[B117] Schipper-Krom S. Juenemann K. Jansen A. H. Wiemhoefer A. Van Den Nieuwendijk R. Smith D. L. (2014). Dynamic recruitment of active proteasomes into polyglutamine initiated inclusion bodies. *FEBS Lett.* 588 151–159 10.1016/j.febslet.2013.11.023 24291262

[B118] Seifert U. Bialy L. P. Ebstein F. Bech-Otschir D. Voigt A. Schröter F. (2010). Immunoproteasomes preserve protein homeostasis upon interferon-induced oxidative stress. *Cell* 142 613–624 10.1016/j.cell.2010.07.036 20723761

[B119] Seo H. Sonntag K. Isacson O. (2004). Generalized brain and skin proteasome inhibition in Huntington’s disease. *Ann. Neurol.* 56 319–328 10.1002/ana.20207 15349858

[B120] Sharma R. Fischer M. T. Bauer J. Felts P. A. Smith K. J. Misu T. (2010). Inflammation induced by innate immunity in the central nervous system leads to primary astrocyte dysfunction followed by demyelination. *Acta Neuropathol.* 120 223–236 10.1007/s00401-010-0704-z 20532539 PMC2892605

[B121] Sheng J. G. Mrak R. E. Griffin W. S. (1997). Neuritic plaque evolution in Alzheimer’s disease is accompanied by transition of activated microglia from primed to enlarged to phagocytic forms. *Acta Neuropathol.* 94 1–5 10.1007/s004010050664 9224523

[B122] Shin J. Y. Fang Z. H. Yu Z. X. Wang C. E. Li S. H. Li X. J. (2005). Expression of mutant huntingtin in glial cells contributes to neuronal excitotoxicity. *J. Cell Biol.* 171 1001–1012 10.1083/jcb.200508072 16365166 PMC2171327

[B123] Shults C. W. (2006). Lewy bodies. *Proc. Natl. Acad. Sci. U.S.A.* 103 1661–1668 10.1073/pnas.0509567103 16449387 PMC1413649

[B124] Sijts E. J. Kloetzel P. M. (2011). The role of the proteasome in the generation of MHC class I ligands and immune responses. *Cell. Mol. Life Sci.* 68 1491–1502 10.1007/s00018-011-0657-y 21387144 PMC3071949

[B125] Simpson J. E. Ince P. G. Lace G. Forster G. Shaw P. J. Matthews F. (2010). Astrocyte phenotype in relation to Alzheimer-type pathology in the ageing brain. *Neurobiol. Aging* 31 578–590 10.1016/j.neurobiolaging.2008.05.015 18586353

[B126] Smith D. M. Kafri G. Cheng Y. Ng D. Walz T. Goldberg A. L. (2005). ATP binding to PAN or the 26S ATPases causes association with the 20S proteasome, gate opening, and translocation of unfolded proteins. *Mol. Cell.* 20 687–698 10.1016/j.molcel.2005.10.019 16337593

[B127] Sofroniew M. V. Vinters H. V. (2010). Astrocytes: biology and pathology. *Acta Neuropathol.* 119 7–35 10.1007/s00401-009-0619-8 20012068 PMC2799634

[B128] Solano R. M. Casarejos M. J. Menéndez-Cuervo J. Rodriguez-Navarro J. A. García De Yébenes J. Mena M. A. (2008). Glial dysfunction in Parkin null mice: effects of aging. *J. Neurosci.* 28 598–611 10.1523/JNEUROSCI.4609-07.2008 18199761 PMC6670347

[B129] Sosunov A. A. Guilfoyle E. Wu X. Mckhann G. M. 2nd Goldman J. E. (2013). Phenotypic conversions of “protoplasmic” to “reactive” astrocytes in Alexander disease. *J. Neurosci.* 33 7439–7450 10.1523/JNEUROSCI.4506-12.2013 23616550 PMC3694721

[B130] Stefanova N. Kaufmann W. A. Humpel C. Poewe W. Wenning G. K. (2012). Systemic proteasome inhibition triggers neurodegeneration in a transgenic mouse model expressing human alpha-synuclein under oligodendrocyte promoter: implications for multiple system atrophy. *Acta Neuropathol.* 124 51–65 10.1007/s00401-012-0977-5 22491959 PMC3377902

[B131] Stohwasser R. Giesebrecht J. Kraft R. Müller E. Häusler K. G. Kettenmann H. (2000). Biochemical analysis of proteasomes from mouse microglia: induction of immunoproteasomes by interferon-γ and lipopolysaccharide. *Glia* 29 355–365 10.1002/(SICI)1098-1136(20000215)29:4<355::AID-GLIA6>3.0.CO;2-4 10652445

[B132] Tai Y. F. Pavese N. Gerhard A. Tabrizi S. J. Barker R. A. Brooks D. J. (2007). Microglial activation in presymptomatic Huntington’s disease gene carriers. *Brain* 130 1759–1766 10.1093/brain/awm044 17400599

[B133] Tambuyzer B. R. Ponsaerts P. Nouwen E. J. (2009). Microglia: gatekeepers of central nervous system immunology. *J. Leukoc. Biol.* 85 352–370 10.1189/jlb.0608385 19028958

[B134] Tanaka Y. Engelender S. Igarashi S. Rao R. K. Wanner T. Tanzi R. E. (2001). Inducible expression of mutant alpha-synuclein decreases proteasome activity and increases sensitivity to mitochondria-dependent apoptosis. *Hum. Mol. Genet.* 10 919–926 10.1093/hmg/10.9.919 11309365

[B135] Tang G. Perng M. D. Wilk S. Quinlan R. Goldman J. E. (2010). Oligomers of Mutant Glial Fibrillary Acidic Protein (GFAP) inhibit the proteasome system in Alexander disease astrocytes, and the small heat shock protein aB-crystallin reverses the inhibition. *J. Biol. Chem.* 285 10527–10537 10.1074/jbc.M109.067975 20110364 PMC2856260

[B136] Tang G. Xu Z. Goldman J. E. (2006). Synergistic effects of the Sapk/Jnk and the proteasome pathway on glial fibrillary acidic protein (Gfap) accumulation in Alexander disease. *J. Biol. Chem.* 281 38634–38643 10.1074/jbc.M604942200 17038307

[B137] Tao J. Wu H. Lin Q. Wei W. Lu X. Cantle J. P. (2011). Deletion of astroglial dicer causes non-cell-autonomous neuronal dysfunction and degeneration. *J. Neurosci.* 31 8306–8319 10.1523/JNEUROSCI.0567-11.2011 21632951 PMC3500097

[B138] Tashiro Y. Urushitani M. Inoue H. Koike M. Uchiyama Y. Komatsu M. (2012). Motor neuron-specific disruption of proteasomes, but not autophagy, replicates amyotrophic lateral sclerosis. *J. Biol. Chem.* 287 42984–42994 10.1074/jbc.M112.417600 23095749 PMC3522293

[B139] Thompson L. M. Aiken C. T. Kaltenbach L. S. Agrawal N. Illes K. Khoshnan A. (2009). Ikk phosphorylates huntingtin and targets it for degradation by the proteasome and lysosome. *J. Cell Biol.* 187 1083–1099 10.1083/jcb.200909067 20026656 PMC2806289

[B140] Tofaris G. K. Razzaq A. Ghetti B. Lilley K. S. Spillantini M. G. (2003). Ubiquitination of a-Synuclein in Lewy bodies is a pathological event not associated with impairment of proteasome function. *J. Biol. Chem.* 278 44405–44411 10.1074/jbc.M308041200 12923179

[B141] Tong X. Ao Y. Faas G. C. Nwaobi S. E. Xu J. Haustein M. D. (2014). Astrocyte Kir4.1 ion channel deficits contribute to neuronal dysfunction in Huntington’s disease model mice. *Nat. Neurosci.* 17 694–703 10.1038/nn.3691 24686787 PMC4064471

[B142] Trotti D. Rolfs A. Danbolt N. C. Brown R. H. Jr. Hediger M. A. (1999). Sod1 mutants linked to amyotrophic lateral sclerosis selectively inactivate a glial glutamate transporter. *Nat. Neurosci.* 2 427–433 10.1038/8091 10321246

[B143] Truant R. Atwal R. S. Desmond C. Munsie L. Tran T. (2008). Huntington’s disease: revisiting the aggregation hypothesis in polyglutamine neurodegenerative diseases. *FEBS J.* 275 4252–4262 10.1111/j.1742-4658.2008.06561.x 18637947

[B144] Tsai Y. C. Fishman P. S. Thakor N. V. Oyler G. A. (2003). Parkin facilitates the elimination of expanded polyglutamine proteins and leads to preservation of proteasome function. *J. Biol. Chem.* 278 22044–22055 10.1074/jbc.M212235200 12676955

[B145] Tseng B. P. Green K. N. Chan J. L. Blurton-Jones M. Laferla F. M. (2008). Abeta inhibits the proteasome and enhances amyloid and tau accumulation. *Neurobiol. Aging* 29 1607–1618 10.1016/j.neurobiolaging.2007.04.014 17544172 PMC2664168

[B146] Tydlacka S. Wang C. Wang X. Li S. Li X. (2008). Differential activities of the ubiquitin–proteasome system in neurons versus Glia may account for the preferential accumulation of misfolded proteins in neurons. *J. Neurosci.* 28 13285–13295 10.1523/JNEUROSCI.4393-08.2008 19052220 PMC2662777

[B147] Van Den Berge S. A. Middeldorp J. Zhang C. E. Curtis M. A. Leonard B. W. Mastroeni D. (2010). Longterm quiescent cells in the aged human subventricular neurogenic system specifically express Gfap-delta. *Aging Cell* 9 313–326 10.1111/j.1474-9726.2010.00556.x 20121722

[B148] van Leeuwen F. W. De Kleijn D. P. V. Van Den Hurk H. H. Neubauer A. Sonnemans M. A. F. Sluijs J. A. (1998). Frameshift mutants of ß amyloid precursor protein and Ubiquitin-B in Alzheimer’s and Down patients. *Science* 279 242–247 10.1126/science.279.5348.242 9422699

[B149] van Tijn P. De Vrij F. M. S. Schuurman K. G. Dantuma N. P. Fischer D. F. Van Leeuwen F. W. (2007). Dose-dependent inhibition of proteasome activity by a mutant ubiquitin associated with neurodegenerative disease. *J. Cell Sci.* 120 1615–1623 10.1242/jcs.03438 17405812

[B150] Vilchez D. Boyer L. Morantte I. Lutz M. Merkwirth C. Joyce D. (2012). Increased proteasome activity in human embryonic stem cells is regulated by Psmd11. *Nature* 489 304–308 10.1038/nature11468 22972301 PMC5215918

[B151] Walker A. K. Daniels C. M. Goldman J. E. Trojanowski J. Q. Lee V. M. Messing A. (2014). Astrocytic Tdp-43 pathology in Alexander disease. *J. Neurosci.* 34 6448–6458 10.1523/JNEUROSCI.0248-14.2014 24806671 PMC4012304

[B152] Walker F. O. (2007). Huntington’s disease. *Lancet* 369 218–228 10.1016/S0140-6736(07)60111-1 17240289

[B153] Wang L. Lin F. Wang J. Wu J. Han R. Zhu L. (2012). Expression of mutant N-terminal huntingtin fragment (htt552-100Q) in astrocytes suppresses the secretion of Bdnf. *Brain Res.* 1449 69–82 10.1016/j.brainres.2012.01.077 22410294

[B154] Wyss-Coray T. Loike J. D. Brionne T. C. Lu E. Anankov R. Yan F. (2003). Adult mouse astrocytes degrade amyloid-beta *in vitro* and *in situ*. *Nat. Med.* 9 453–457 10.1038/nm838 12612547

[B155] Yamamoto A. Friedlein A. Imai Y. Takahashi R. Kahle P. J. Haass C. (2005). Parkin phosphorylation and modulation of Its E3 ubiquitin ligase activity. *J. Biol. Chem.* 280 3390–3399 10.1074/jbc.M407724200 15557340

[B156] Yan L. J. Xiao M. Chen R. Cai Z. (2013). Metabolic dysfunction of astrocyte: an initiating factor in beta-amyloid pathology? *Aging Neurodegener*. 1 7–14 24443714 PMC3891850

[B157] Zhang D. Hu X. Qian L. O’callaghan J. P. Hong J. S. (2010). Astrogliosis in CNS pathologies: is there a role for microglia? *Mol. Neurobiol*. 41 232–241 10.1007/s12035-010-8098-4 20148316 PMC3629545

[B158] Zhang H. Tan C. F. Mori F. Tanji K. Kakita A. Takahashi H. (2008). Tdp-43-immunoreactive neuronal and glial inclusions in the neostriatum in amyotrophic lateral sclerosis with and without dementia. *Acta Neuropathol.* 115 115–122 10.1007/s00401-007-0285-7 17786458

[B159] Zhao X. Yang J. (2010). Amyloid-beta peptide is a substrate of the human 20S proteasome. *ACS Chem. Neurosci.* 1 655–660 10.1021/cn100067e 21116456 PMC2992454

[B160] Zheng J. Dasgupta A. Bizzozero O. A. (2012). Changes in 20S subunit composition are largely responsible for altered proteasomal activities in experimental autoimmune encephalomyelitis. *J. Neurochem.* 121 486–494 10.1111/j.1471-4159.2012.07699.x 22353035 PMC3323733

